# Multitarget evaluation of 4-substituted 7-hydroxycoumarin derivatives: anticancer activity, topoisomerase I inhibition, and interaction with human serum albumin

**DOI:** 10.1007/s00210-026-05062-w

**Published:** 2026-02-12

**Authors:** Adrián Gucký, Martin Majerník, Slávka Hamuľaková, Katarzyna E. Nowak, Rastislav Jendželovský, Peter Fedoročko, Mária Kožurková

**Affiliations:** 1 Department of Biochemistry, Institute of Chemistry, Faculty of Science, P. J. Šafárik Universityin Košice, Moyzesova 11, 040 01 Košice, Slovak Republic; 2 Department of Cell Biology, Institute of Biology and Ecology, Faculty of Science, P. J. Šafárik Universityin Košice, Šrobárova 2, 040 01 Košice, Slovak Republic; 3 Department of Organic Chemistry, Institute of Chemistry, Faculty of Science, P. J. Šafárik Universityin Košice, Moyzesova 11, 040 01 Košice, Slovak Republic; 4 Department of Oncobiology and Epigenetics, Faculty of Biology and Environmental Protection, University of Lodz, Pomorska 141/143, 90-236, Lodz, Poland

**Keywords:** Coumarin, HSA, Topoisomerase I, A549

## Abstract

Coumarins are known to provide promising scaffolds for the development of new anticancer drugs, yet their multitarget biological profiles remain insufficiently explored. This study presents a comprehensive evaluation of four newly synthesized 4-substituted 7-hydroxycoumarin derivatives **C1**–**C4**, highlighting their combined antiproliferative, enzyme-inhibitory, and pharmacokinetic properties. The compounds were tested for their cytotoxic effects on A549 lung carcinoma cells and CCD-18Co fibroblasts, inhibition of topoisomerase I (Topo I), and binding interactions with human serum albumin (HSA). Derivatives **C1**, **C2**, and **C4** showed selective suppression of A549 metabolic activity and proliferation, while exhibiting minimal toxicity toward non-cancerous fibroblasts. All compounds inhibited Topo I to varying degrees, with **C1** displaying the highest potency, indicating that specific hydroxyl group arrangements are crucial for enzyme inhibition. Fluorescence spectroscopy and molecular docking revealed moderate to high HSA affinity (10^4^–10^6^ M^−1^) and predominantly single-site binding, supporting their favorable plasma transport potential. Thermodynamic analysis showed distinct interaction patterns: **C1** and **C2** formed complexes stabilized primarily by hydrogen bonds and van der Waals forces, whereas **C3** and **C4** interacted mainly through hydrophobic forces. Competitive displacement assays identified Sudlow site I as the principal binding region, although molecular docking studies also suggested the possible involvement of site III. Overall, this work introduces a novel series of coumarin derivatives with a clearly defined multitarget anticancer profile which combines selective cytotoxicity, effective Topo I inhibition, and strong pharmacokinetic characteristics, thereby underscoring the potential of these derivatives as lead structures for further anticancer drug development.

**Supplementary Information:**

The online version contains supplementary material available at 10.1007/s00210-026-05062-w.

## Introduction

The majority of anticancer drugs currently used in clinical practice are associated with significant side effects, and increasing evidence suggests that resistance to existing therapies may limit their long-term effectiveness. These challenges highlight the need to discover new biologically active compounds that can provide strong therapeutic effects with minimal toxicity. Recent efforts in the field of medicinal chemistry have focused on the development of multitarget small-molecule anticancer agents that combine cytotoxic activity with well-defined molecular mechanisms. Several newly reported scaffolds, such as imidazole-based hybrids (Alsirhani et al. 2025), sulfadiazine-derived epidermal growth factor receptor (EGFR) inhibitors (El-Lateef et al. 2024), or thiophene hydrazides with dual EGFR/tubulin inhibition (Aboelez et al. 2025), have demonstrated the benefits of integrating antiproliferative testing with mechanistic assays and in silico profiling. Coumarins, naturally occurring benzopyrone derivatives which are found relatively commonly in various plant species, have emerged as promising candidates for the development of novel anticancer agents. These compounds display a wide variety of biological and pharmacological activities and are already employed in medicine, cosmetics, and fluorescence-based studies (Dadashpour and Emami 2015; Salem et al. 2016; Stefanachi et al. 2018).

Coumarin derivatives are generally easy and cost-effective to synthesize, and this has allowed researchers to conduct extensive studies to identify their wide range of pharmacological effects (Bisi et al. 2017). These activities range from anticancer effects (such as inducing apoptosis or inhibiting the assembly of microtubules) and antimicrobial properties to antioxidant and anticoagulant activity, as has been observed with warfarin and other 4-hydroxycoumarins through the inhibition of vitamin K epoxide reductase. Additional effects include the inhibition of enzymes such as cholinesterases, monoamine oxidases, topoisomerases, carboanhydrases, protein kinases, and histone deacetylases (Al-Warhi et al. 2020; Bisi et al. 2017; Dadashpour and Emami 2015; Dandriyal et al. 2016; Delgado et al. 2018; Gacche and Jadhav 2012; Morsy et al. 2017; Pan et al. 2022; Salem et al. 2016; Sandhu et al. 2014; Yadav et al. 2024). Significantly, coumarins rarely display cardiotoxic, nephrotoxic, or hepatotoxic effects, and this important distinction has contributed to the increased interest in the compounds for research into new chemotherapy approaches (Dadashpour and Emami 2015; Dandriyal et al. 2016).

Various coumarin derivatives with anticancer potential have been synthesized, mainly through substitutions at positions 3, 4, 7, and 8 or by conjugation with other heterocyclic compounds or an additional coumarin moiety (Dadashpour and Emami 2015). Maximum cytotoxicity is often achieved with substitutions at positions 4 and 7, as well as O-substitution on the -OH group of 7-hydroxycoumarins (Dadashpour and Emami 2015; Dandriyal et al. 2016; Bisi et al. 2017).

Topoisomerase I (Topo I) is a crucial enzyme that relaxes DNA supercoiling during transcription and replication (Durand-Dubief et al. 2011; Pommier et al. 2010; Wang 2002). Many cancer cells overexpress Topo I to sustain rapid proliferation, and the suppression or inhibition of Topo I has thus emerged as a potential therapeutic approach. Certain coumarin derivatives have been found to inhibit Topo I, leading to DNA damage and cell death (Yadav et al. 2024). Among the most potent inhibitors are 4-hydroxycoumarins, such as warfarin, which primarily act as anticoagulants. Other derivatives have been designed to selectively stabilize the Topo I-DNA complex, preventing religation in a manner similar to that exercised by camptothecin (CPT). Hybrid molecules, including coumarin-quinoline, tacrine-coumarin, coumarin-imidazole, and coumarin-thiadiazole derivatives, have also demonstrated strong Topo I inhibition (Al-Warhi et al. 2020; Fotopoulos and Hadjipavlou-Litina 2020; Konkoľová et al. 2021; Yadav et al. 2024).

Human serum albumin (HSA), the most abundant protein in plasma, plays a central role in drug transport and pharmacokinetics. Ligands bind primarily at Sudlow sites I and II or at a third site within subdomain IB, with hydrophobic interactions, hydrogen bonding, and van der Waals forces influencing the binding affinity, solubility, stability, metabolism, and free drug availability (Yue et al. 2018; Zsila 2013). Strong binding can prolong the half-life of agents, but this also reduces the availability of free drugs; in contrast, weak binding can accelerate clearance and increase toxicity. Fluorescence spectroscopy, circular dichroism, and molecular docking simulations are commonly used to study these interactions (Chamlagai et al. 2024; Harder and Thürmann 1996; Khan et al. 2019; Shobini et al. 2001; Yang et al. 2024).

In this study, we investigate the antiproliferative effects of newly synthesized coumarin derivatives (**C1**–**C4**) on the A549 and CCD-18Co cell lines, alongside their effects on Topo I activity. Fluorescence spectroscopy and molecular docking studies were employed to determine the binding constants, preferred binding sites, thermodynamic parameters, and interaction modes of the coumarin–HSA complexes.

## Materials and methods

### Reagents

**Fig. 1:**
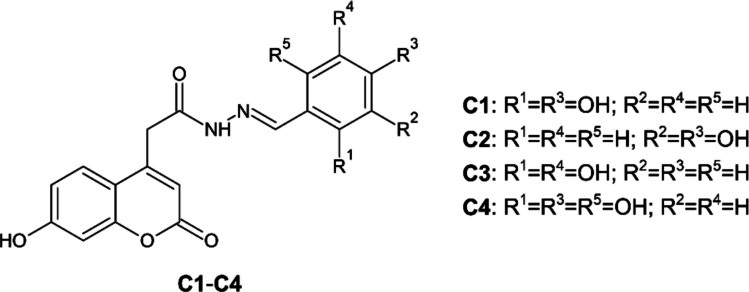
Chemical structures of coumarin derivatives **C1**–**C4** (Hamuľaková et al. 2025)

All reagents were obtained from standard commercial suppliers and used as delivered, without additional purification. Coumarin derivatives **C1**–**C4** (Table 1, Fig. 1) were synthesized according to the procedure reported by Hamuľaková et al. (2025). For experimental use, the solid compounds were dissolved in dimethyl sulfoxide (DMSO; Merck, Prague, Czech Republic) to prepare stock solutions of 10 mM, which were subsequently diluted to the required concentrations. Human serum albumin (HSA, fatty acid free; Sigma-Aldrich, St. Louis, MO, USA) was dissolved in phosphate-buffered saline (PBS; 10 mM, pH 7.4) at 40 mg.mL^−1^. The concentration of the HSA solution was verified spectrophotometrically at 280 nm using the molar extinction coefficient ε_280_ = 36 850 M^−1^.cm^−1^ (Chatterjee et al. 2012).

**Table 1:** Systematic names and molar mass of compounds **C1**–**C4** (Hamuľaková et al. 2025)

Compound	IUPAC name	M_r_ (g.mol^−1^)
C1	*N*´-[(*E*)-(2,4-dihydroxyphenyl)methylidene]−2-(7-hydroxy-2-oxo-2*H*-chromen-4-yl)acetohydrazide	354.31
C2	*N*´-[(*E*)-(3,4-dihydroxyphenyl)methylidene]−2-(7-hydroxy-2-oxo-2*H*-chromen-4-yl)acetohydrazide	354.31
C3	*N*´-[(*E*)-(2,5-dihydroxyphenyl)methylidene]−2-(7-hydroxy-2-oxo-2*H*-chromen-4-yl)acetohydrazide	354.31
C4	*N*´-[(*E*)-(2,4,6-trihydroxyphenyl)methylidene]−2-(7-hydroxy-2-oxo-2*H*-chromen-4-yl)acetohydrazide	370.31

### Cell cultures

The human lung adenocarcinoma epithelial cell line A549 (RRID: CVCL_0023) and the non-tumorigenic colon fibroblast line CCD-18Co (RRID: CVCL_2379) were obtained from the American Type Culture Collection (ATCC, Rockville, MD, USA). The A549 cells were cultivated in a RPMI-1640 medium (Sigma-Aldrich, St. Louis, MO, USA), with passaging performed twice weekly, while the CCD-18Co cells were cultivated in MEM medium (Biosera, Nuaillé, France) and passaged once per week. Both culture media were supplemented with 10% fetal bovine serum (FBS; Biosera, Nuaillé, France), together with a 1% antibiotic–antimycotic solution (100 ×) and gentamicin at 50 μg.mL^−1^ (Biosera, Nuaillé, France). Cell cultures were stored at 37 °C under 5% CO₂ and 95% relative humidity, and the media were refreshed twice weekly.

### MTT assays

MTT assays were performed using the previously reported method (Kleban et al. 2007; Křikavová et al. 2023) to evaluate the changes in the metabolic activity of cells (5000 cells per well) following 24 h and 48 h of treatment with derivatives (**C1**–**C4**) at concentrations of 5, 25, 50, and 75 µM.

### Cell proliferation assay

The antiproliferative effect of the derivatives was also investigated by monitoring their label-free proliferation in real time using the IncuCyte™ ZOOM live-cell imaging system (Essen BioScience, Ann Arbor, MI, USA). Cells were seeded in 96-well plates (TPP, Trasadingen, Switzerland) at 5000 cells per well and allowed to adhere for 24 h. The derivatives were then added at concentrations of 5, 25, 50, and 75 µM. Cell confluence was recorded every 2 h over a period of 74 h using IncuCyte™ ZOOM software, and the results were evaluated as percentages of the confluence of the untreated control and each experimental group.

### Statistical analysis

All quantitative results from MTT and proliferation assays represent the mean ± standard deviation (SD) of three independent replicates. Statistical significance was determined using one-way ANOVA followed by Tukey’s post hoc test. Specific significance levels are reported in each figure legend.

### Topoisomerase I relaxation assay

The inhibitory activity of **C1**–**C4** against human topoisomerase I (*h*Topo I; Inspiralis, Norwich, UK; 1.0 U/sample) was tested using a previously published procedure (Janovec et al. 2025) with camptothecin (CPT) being used as a positive control.

### DNA unwinding assay

DNA unwinding was evaluated using wheat germ topoisomerase I (*wg*Topo I; Inspiralis, Norwich, UK; 2.5 U/sample) with supercoiled and relaxed pBR322 DNA (0.5 µg/sample) as substrates and ethidium bromide as a positive control. The assays were conducted according to a previously described protocol (Janovec et al. 2025).

### Steady-state fluorescence spectroscopy

Fluorescence data were collected on a Varian Cary Eclipse spectrophotometer (Varian Medical Systems, Sydney, Australia) at 15, 20, 25, 30, and 37 °C. Samples were prepared in a PBS buffer (10 mM, pH 7.4) in a 1 × 1 cm quartz cuvette (Hellma, Müllheim, Germany). The excitation wavelength was set to 280 nm, and the emission spectra were collected between 290 and 500 nm, with excitation and emission slit widths of 5 and 10 nm, respectively. Spectra were measured at 2.1 µM HSA with increasing concentrations of **C1**–**C4** (0–9.9 µM). Each measurement was performed in triplicate, and the results are expressed as the mean ± SD.

### Synchronous fluorescence spectroscopy

Synchronous fluorescence spectra were recorded at 25 °C. Two scanning intervals (Δλ = λ_em_–λ_ex_) were applied: Δλ = 15 nm to monitor tyrosine residues and Δλ = 60 nm for tryptophan. Measurements were carried out in PBS buffer (10 mM, pH 7.4) using a 1 × 1 cm quartz cuvette. Excitation and emission slit widths were set at 10 nm. The initial HSA concentration was 2.1 µM, with stepwise additions of **C1**–**C4** (0–29.1 µM).

### Three-dimensional fluorescence spectroscopy

Three-dimensional fluorescence spectra were obtained at 25 °C using a 1 × 1 cm quartz cuvette. Samples of free HSA (2.6 µM) and HSA-coumarin complexes (1:1 ratio) were analyzed in PBS buffer (10 mM, pH 7.4). Excitation was scanned from 200 to 350 nm in 10-nm increments, and emission was monitored between 200 and 600 nm. Slit widths were set at 5 nm (excitation) and 10 nm (emission).

### Competitive displacement assays

Binding site identification was conducted using site markers: warfarin (site I), ibuprofen (site II), and digitoxin (site III). Experiments were performed at 25 °C in PBS buffer (10 mM, pH 7.4) using a 1 × 1 cm cuvette. HSA-coumarin complexes (2.6 µM, 1:1 ratio) were excited at 280 nm, and emission was recorded from 290 to 500 nm with excitation and emission slits set to 5 and 10 nm, respectively. Site markers were added stepwise to achieve molar ratios from 1:1 up to 1:10. Reverse titrations were also carried out, with HSA-site marker complexes (2.1 µM, 1:1) titrated with increasing concentrations of **C1**–**C4 **(0–9.9 µM). All assays were performed in triplicate, and data are given as mean ± SD.

### Molecular modelling and docking simulations

Molecular modelling of the ligands in the Gaussian 03 program (Frisch et al. 2004) and the GaussView 4.1.2 package (Dennington et al. 1997–2007) was performed using the same methods as in previous study (Konkoľová et al. 2024). The crystal structure of the ternary DNA cleavage complex of hTopo I (PDB ID: 1T8I (Staker et al. 2005a, b)) was downloaded from the Protein Data Bank (Berman et al. 2000). The hTopo I-DNA complex molecule was prepared for docking in the ADT 1.5.6rc3 software package (Morris et al. 2009; Sanner 1999). Simulations of flexible ligand docking were carried out in AutoDock 4.2 using the Lamarckian genetic algorithm (Morris et al. 1998). The grid box was set as reported by Janovec et al. (2025). The structure of HSA (PDB ID: 1AO6 (Sugio et al. 1998, 1999)) was prepared for docking in the ADT 1.5.6rc3 software package (Morris et al. 2009; Sanner 1999). The grid box was set and simulations of ligands docking to HSA (in AutoDock 4.2 (Morris et al. 1998)) were performed as previously (Konkoľová et al. 2024). The top score positions of each ligand were selected to generate a representative complex model. Discovery Studio Visualizer (Biovia, Dassault Systems 2020) was used invisualization and analysis of the results.

## Results and discussion

### Metabolic activity

**Fig. 2:**
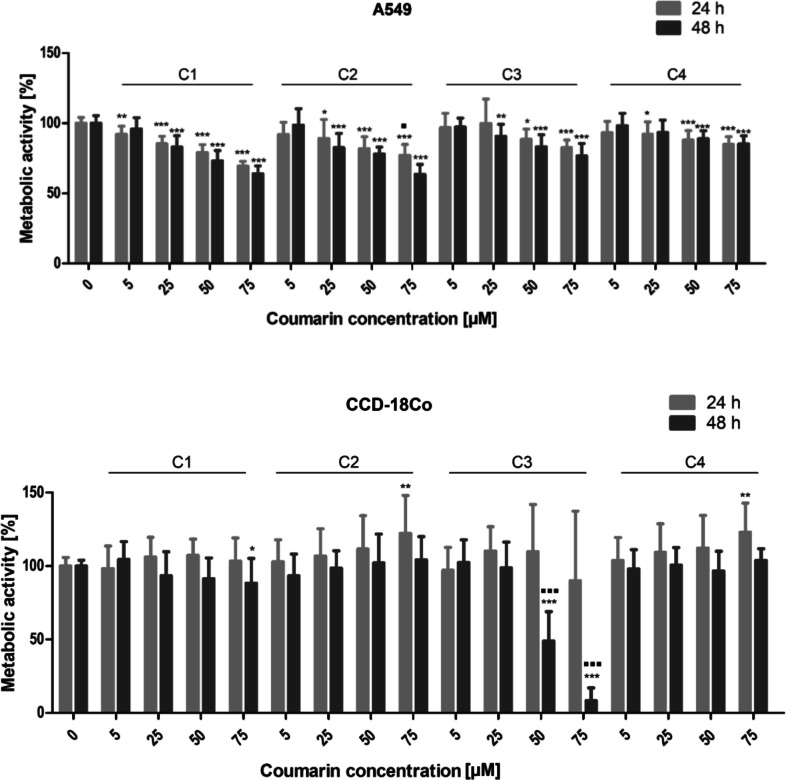
Effect of compounds **C1**–**C4** on the metabolic activity of the A549 and CCD-18Co cell lines after 24 h and 48 h of incubation with varying concentrations of the studied compounds. The experimental groups affected by the tested coumarins were compared with the control group (* *p* < 0.05, ** *p* < 0.01, *** *p* < 0.001), and the experimental groups analyzed after 24 h were compared with the experimental groups analyzed after 48 h (▪ *p* < 0.05, ▪▪ *p* < 0.01, ▪▪▪ *p* < 0.001)

The metabolic activity of the A549 and CCD-18Co cell lines was assessed as an overall signal resulting from the number of metabolizing cells and the intensity of their metabolism after exposure to the novel coumarins. The results showed that the cancer cells displayed considerable sensitivity to the activity of the tested coumarins, with all of the novel derivatives inhibiting the metabolism of the A549 cancer cell line (Fig. 2). In the case of the non-cancerous CCD-18Co cell line, a significant inhibition of metabolic activity was observed for derivatives **C3** and **C1**, but only at the highest tested concentration after 48 h (Fig. 2). An increase in metabolic activity after application of coumarins was observed only in the non-cancerous CCD-18Co cell line, specifically for coumarins **C2** and **C4**.

### Analysis of cell proliferation

**Fig. 3:**
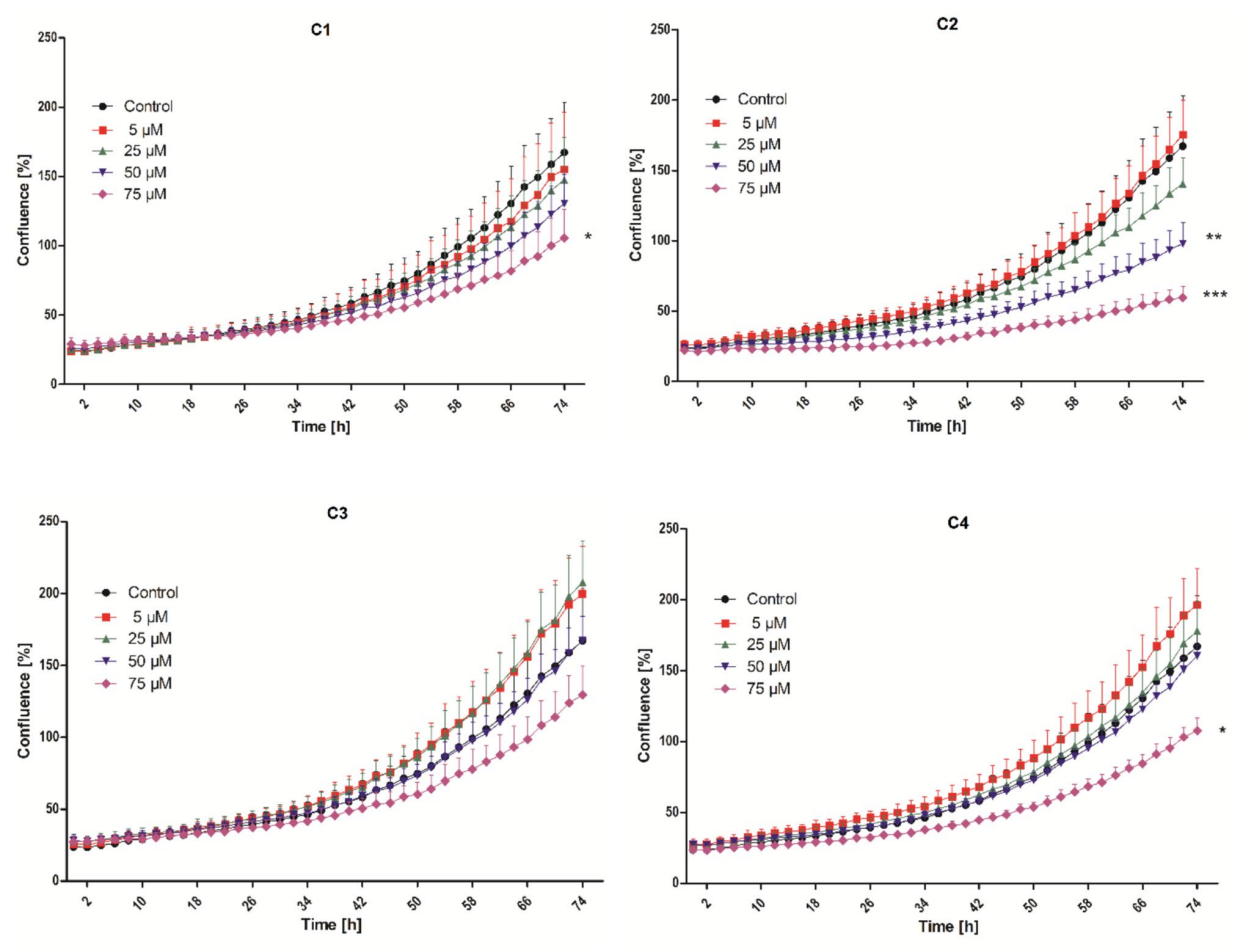
Effect of compounds **C1**–**C4** on the proliferation of A549 cells over a 74-h time interval. The experimental groups affected by the tested coumarins were compared with the control group (* *p* < 0.05, ** *p* < 0.01, *** *p* < 0.001)

The biological effects of the novel coumarins were investigated more fully through a prolonged test aimed at analyzing their impact on the proliferation of both cancerous and non-cancerous cells. The results showed that the novel derivatives had a less pronounced effect on cell proliferation than on metabolic activity. For the A549 cell line, an inhibition of proliferation was observed in the experimental groups treated with coumarins **C1**, **C2**, and **C4**, with a marked decrease in proliferation after the application of **C2** at concentrations of 50 µM and 75 µM and of **C1** and **C4** at a concentration of 75 µM, findings that correlate with the results of the metabolic activity analysis (Fig. 3). The results further show that no significant stimulation of A549 proliferation was observed across the tested concentration range.

**Fig. 4:**
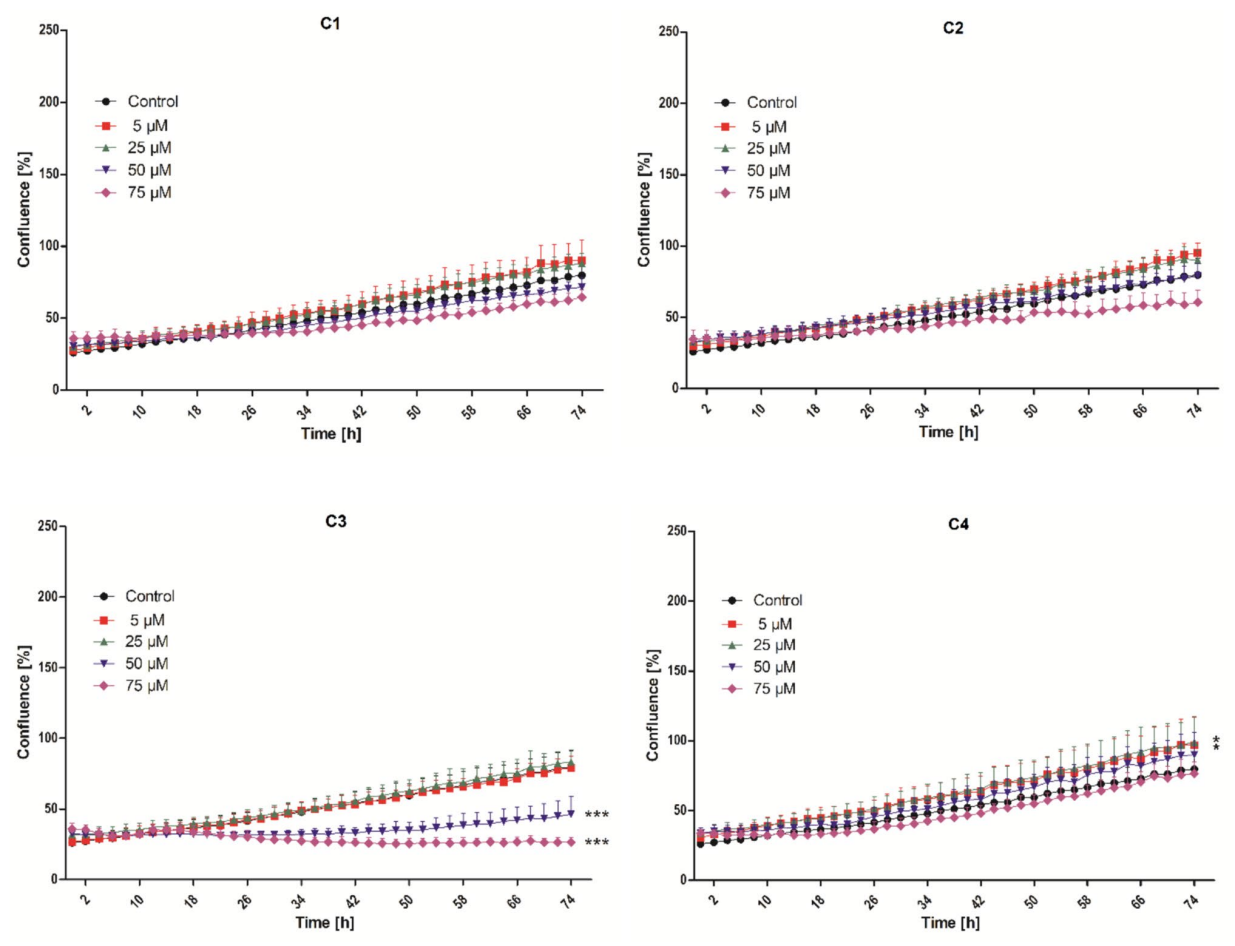
Effect of compounds **C1**–**C4** on the proliferation of CCD-18Co cells throughout a 74-h time interval. The experimental groups affected by tested coumarins were compared with the control group (* *p* < 0.05, ** *p* < 0.01, *** *p* < 0.001)

However, the analysis of the non-cancerous CCD-18Co cell line revealed the stimulation of cell proliferation in the experimental groups treated with derivative **C4** at concentrations of 5 µM and 25 µM (Fig. 4). The reduced proliferative status of non-cancerous CCD-18Co cells in the experimental groups treated with coumarin **C3** at concentrations of 50 µM and 75 µM correlates with the results of the metabolic activity analysis, in which inhibition was also observed. In the case of coumarins **C1** and **C2**, no effect on cell proliferation in the CCD-18Co cells line was observed.

The slight increase in proliferative activity observed for **C4** in CCD-18Co fibroblasts at low concentrations may reflect a mild hormetic response, a phenomenon which has often been observed in polyhydroxylated coumarins and structurally related phenolic compounds. These molecules can transiently enhance mitochondrial activity or activate cytoprotective pathways when present at subtoxic levels, while higher concentrations produce inhibitory effects (Gacche and Jadhav 2012; Salem et al. 2016). Given the trihydroxy substitution of derivative **C4**, the possibility of such an adaptive metabolic response is a plausible explanation, although further research will be required to confirm this hypothesis. It should also be noted that the effect observed in the assay was relatively modest and was confined to non-cancerous fibroblasts, suggesting that the effect may be a physiological adaptation rather than a proliferative hazard.

### Topoisomerase I inhibition activity

**Fig. 5:**
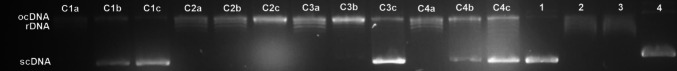
Electrophoretic record of hTopo I inhibition in the presence of compounds **C1**–**C4.** Concentrations: a = 10 µM, b = 50 µM, c = 100 µM. Control samples: 1 = pBR322, 2 = pBR322 + hTopo I, 3 = pBR322 + hTopo I + DMSO, 4 = pBR322 + hTopo I + camptothecin (100 µM). Abbreviations: ocDNA, open circular form of plasmid DNA; rDNA, relaxed form of plasmid DNA; scDNA, supercoiled form of plasmid DNA

Topo I induces the relaxation of supercoiled DNA (scDNA) and the formation of relaxed (rDNA) and open circular (ocDNA) forms. These topoisomers can be separated based on their mobility through the pores of agarose gel, with scDNA migrating at the fastest rate and ocDNA at the slowest, while rDNA forms can be easily distinguished from scDNA and ocDNA. If Topo I remains active, the relaxation of scDNA can be observed, and the resulting electropherogram displays only rDNA and ocDNA forms. In contrast, the inhibition of Topo I activity produces an electropherogram in which only scDNA is visible (Konkoľová et al. 2021). The results of the Topo I relaxation assay in the presence of derivatives **C1**–**C4** at concentrations of 10, 50, and 100 µM are depicted in Fig. 5. The results suggest that compound **C1** is the most effective Topo I inhibitor from the series, exhibiting a level of inhibitory activity comparable to that of CPT at both 50 and 100 µM. A similar inhibitory activity was observed for derivatives **C3** and **C4** at a concentration of 100 µM. Compound **C2** was unable to inhibit the enzymatic relaxation of scDNA effectively even at the highest tested concentration of 100 µM. Interestingly, **C2** is the only compound from the series which lacks a hydroxy group in position 2 of the side chain benzene ring, suggesting that the presence of this feature is crucial in mediating Topo I inhibitory activity.

### Mechanism of topoisomerase I inhibition

**Fig. 6:**
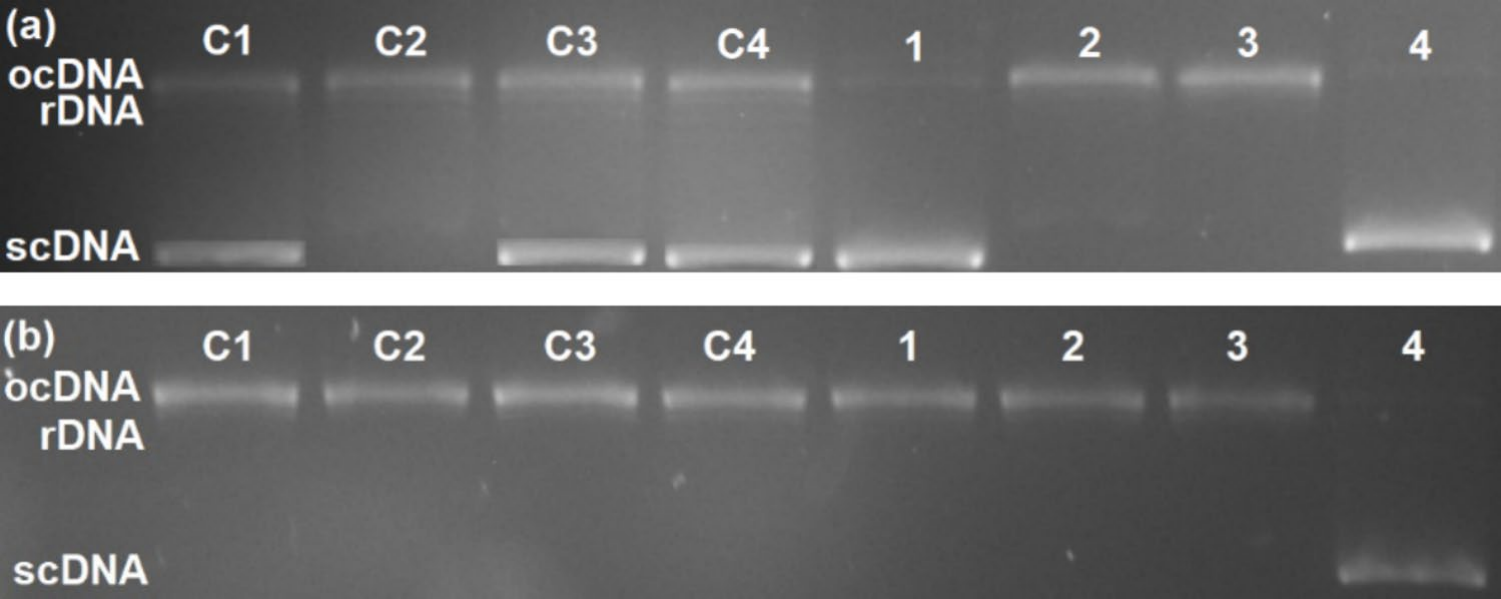
Electrophoretic records of wgTopo I inhibition in the presence of compounds **C1**–**C4** (100 µM). Substrate: (a) supercoiled pBR322, (b) relaxed pBR322. Control samples: 1 = pBR322, 2 = pBR322 + wgTopo I, 3 = pBR322 + wgTopo I + DMSO, 4 = pBR322 + wgTopo I + ethidium bromide (100 µM). Abbreviations: ocDNA, open circular form of plasmid DNA; rDNA, relaxed form of plasmid DNA; scDNA, supercoiled form of plasmid DNA

The mechanism of Topo I inhibition was investigated by performing a DNA unwinding assay using two types of substrates, supercoiled and relaxed pBR322 plasmid DNA, to determine the mode of binding between the coumarin derivatives and DNA. If a compound is capable of DNA intercalation or groove binding, a local unwinding of DNA occurs. When a DNA molecule is nicked and rejoined by a topoisomerase in the presence of such a compound, it converts into a relaxed, underwound form which returns to a supercoiled form once the enzyme is removed. Supercoiled DNA formed under such conditions is therefore indicative of the presence of a DNA binder. Topo I inhibitors incapable of DNA binding simply prevent Topo I from inducing the relaxation of scDNA by interacting directly with the enzyme itself (Palchaudhuri and Hergenrother 2007). Our results (Fig. 6) indicate that coumarin derivatives **C1**–**C4** primarily inhibit Topo I activity through direct binding to the enzyme and can therefore be classified as Topo I inhibitors rather than as DNA intercalators or groove binders. We suggest that if any type of interaction between **C1**–**C4** and the DNA substrate occurs, it is very weak in nature and does not result in the conversion of relaxed plasmids to scDNA (Fig. 6, bottom). The results obtained from the sample set containing supercoiled pBR322 (Fig. 6, top) corroborate the results of the relaxation assay.

### Interaction with HSA

The systemic distribution of pharmaceutical agents in the human body is mainly mediated by their reversible binding to HSA, and a thorough investigation of a potential drug’s interactions with HSA is therefore crucial for understanding the pharmacokinetics and pharmacodynamics of the drug candidate (Meti et al. 2015). Fluorescence spectroscopy is widely used for probing such interactions and monitoring the changes in the microenvironment of HSA fluorophores. Aromatic amino acids (Phe, Tyr, and Trp) are generally responsible for the intrinsic fluorescence of proteins, with Trp having the highest quantum yield and therefore making the largest contribution to the overall fluorescence intensity of the protein, followed by Tyr residues (Konkoľová et al. 2019).

**Fig. 7:**
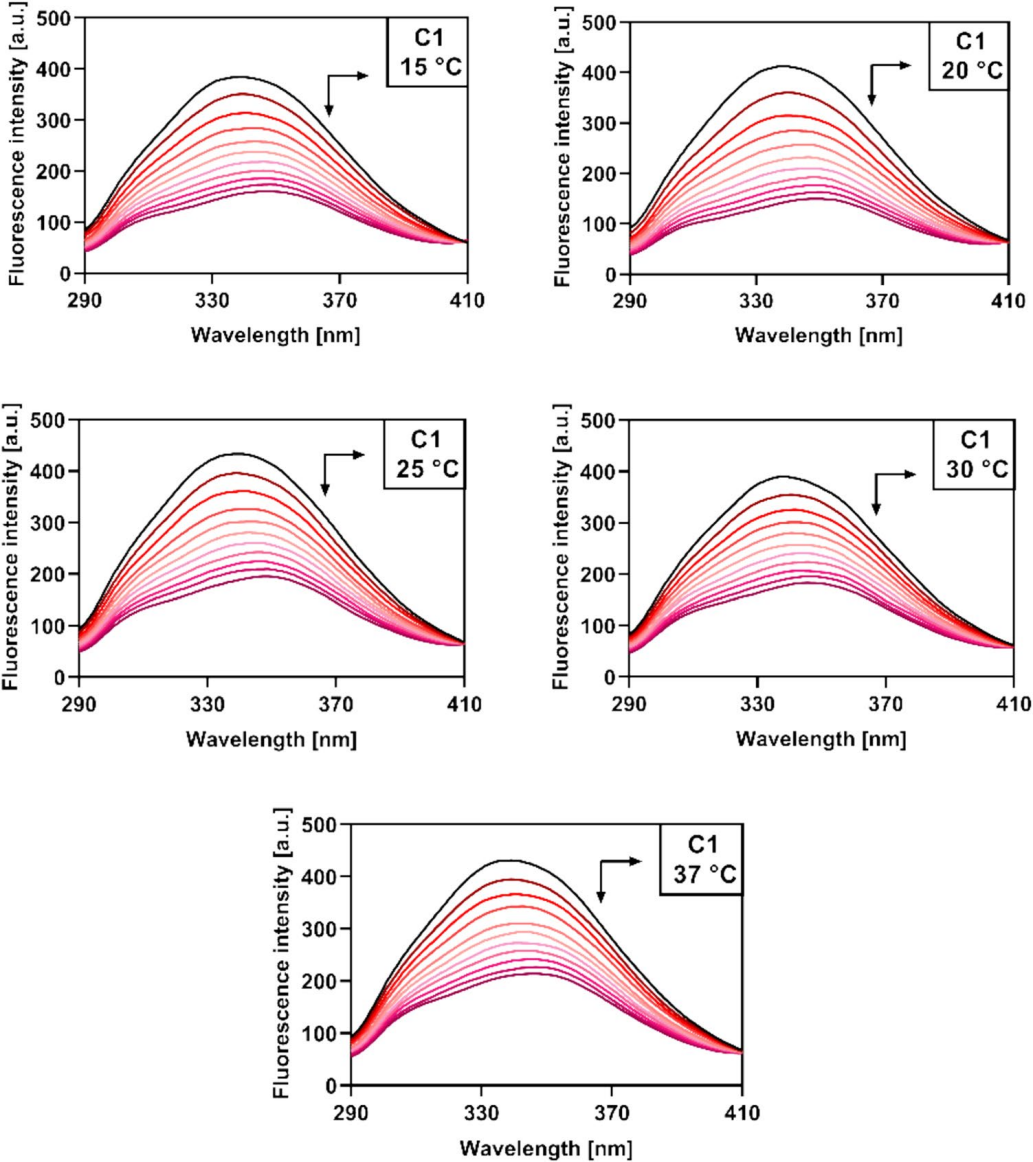
Emission spectra of HSA (2.1 µM) in 10 mM PBS (pH 7.4) upon addition of **C1** (0–9.9 µM) at five different temperatures

Upon excitation at 280 nm, the emission spectra of HSA exhibited a single band with an emission maximum identified at 340 nm. Further additions of coumarin derivatives **C1**–**C4** to the sample led to a gradual quenching of the intrinsic HSA fluorescence in a concentration-dependent manner (Fig. 7, Figs. S1–S3). Fluorescence quenching was observed at each of the tested temperatures and presumably resulted from an ongoing interaction between HSA and the studied compounds, which seem to bind in close proximity to the fluorescent Tyr and Trp residues (Salem et al. 2019). Furthermore, the fluorescence quenching was accompanied by a red shift in the emission maximum wavelength of derivatives **C1** (7 nm), **C2** (7 nm), and **C4** (4 nm), and a blue shift in the case of **C3** (5 nm). These observations indicate that the binding of **C1**–**C4** to HSA also influences the polarity in the microenvironment of the fluorophores adjacent to the corresponding binding site (Zhou et al. 2018); a red shift is typically observed when the microenvironment experiences an increase in polarity and a decrease in hydrophobicity, while a blue shift conversely corresponds to a decrease in polarity and an increase in hydrophobicity (Gökoğlu et al. 2014). On this basis, it can be suggested that the binding of **C3** to HSA is mainly mediated by hydrophobic interactions, while polar interactions, such as hydrogen binding and van der Waals forces, may be involved in the interaction of HSA with the remaining derivatives.

**Fig. 8:**
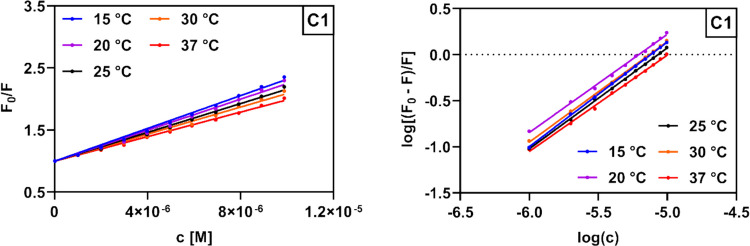
Stern–Volmer plot (left) and logarithmic Stern–Volmer plot (right) for the fluorescence quenching of HSA (2.1 µM) by compound **C1** (0–9.9 µM) at five different temperatures

In general, it is possible to distinguish between two distinct mechanisms of fluorescence quenching—static and dynamic. Static quenching is a result of the formation of a non-fluorescent, ground-state complex between the fluorophore and the quencher, the stability of which is reduced at increasing temperatures (Lakowicz 2010). In contrast, dynamic quenching occurs when the two species are only in contact in the excited state; the elevated temperature results in both faster diffusion and a higher frequency of collisions (Jamshidvand et al. 2018). The quenching process was investigated by plotting corresponding Stern–Volmer graphs (Fig. 8, Fig. S4), and the analysis was performed according to the Stern–Volmer equation (Eq. 1):

1 $${F}_{0}/F={K}_{SV}{c}_{Q}+1$$

where *F*_*0*_ represents the initial fluorescence intensity of HSA, *F* represents the fluorescence intensity after the addition of a corresponding increment of the quencher (**C1**–**C4**), *c*_*Q*_ represents the quencher concentration in the sample, and *K*_*SV*_ is the Stern–Volmer quenching constant. The corresponding *K*_*SV*_ values were obtained from the slopes of the linear Stern–Volmer plots and were found to be around 10^5^ M^−1^ (Table 2). A linear Stern–Volmer plot generally indicates that only a single fluorescence quenching mechanism is present, either static or dynamic (Sarwar et al. 2015). A decrease in the *K*_*SV*_ values with increasing temperature was observed for all of the studied compounds, implying that the fluorescence quenching of HSA by **C1**–**C4** is mediated through a static mechanism involving the formation of a ground-state complex with lower stability at higher temperatures (Lakowicz 2010).

**Table 2:** Stern–Volmer quenching constants (*K*_*SV*_), binding constants (*K*_*b*_*)* and number of binding sites (*n*) of compounds **C1**–**C4** bound to HSA at five different temperatures

Compound	t (°C)	*K*_*SV*_ (× 10^5^ M^−1^)	R^2^*	*K*_*b*_ (× 10^5^ M^−1^)	*n*	R^2^*
**C1**	15	1.31 ± 0.10	0.9972	7.38 ± 0.14	1.15	0.9977
	20	1.23 ± 0.17	0.9954	4.90 ± 0.10	1.11	0.9977
	25	1.16 ± 0.13	0.9976	4.44 ± 0.17	1.09	0.9996
	30	1.08 ± 0.14	0.9944	3.46 ± 0.11	1.09	0.9975
	37	0.97 ± 0.10	0.9956	2.74 ± 0.14	1.08	0.9971
**C2**	15	1.07 ± 0.10	0.9983	6.29 ± 0.21	1.16	0.9983
	20	0.97 ± 0.09	0.9972	2.57 ± 0.14	1.08	0.9992
	25	0.91 ± 0.12	0.9951	1.39 ± 0.10	1.05	0.9966
	30	0.88 ± 0.08	0.9961	0.96 ± 0.07	1.04	0.9983
	37	0.80 ± 0.09	0.9896	0.59 ± 0.11	1.01	0.9945
**C3**	15	1.60 ± 0.13	0.9814	4.44 ± 0.14	1.09	0.9942
	20	1.48 ± 0.12	0.9921	4.62 ± 0.14	1.10	0.9982
	25	1.29 ± 0.10	0.9915	5.11 ± 0.10	1.11	0.9970
	30	1.27 ± 0.07	0.9913	7.87 ± 0.04	1.17	0.9981
	37	1.20 ± 0.07	0.9939	9.60 ± 0.07	1.17	0.9979
**C4**	15	1.84 ± 0.03	0.9906	3.73 ± 0.13	1.06	0.9978
	20	1.81 ± 0.03	0.9964	3.80 ± 0.10	1.06	0.9969
	25	1.69 ± 0.03	0.9930	4.99 ± 0.15	1.13	0.9977
	30	1.46 ± 0.02	0.9934	7.64 ± 0.23	1.14	0.9983
	37	1.39 ± 0.02	0.9905	10.62 ± 0.37	1.17	0.9989

*Correlation coefficient

In order to calculate the binding constants (*K*_*b*_) defining the magnitude of interactions between HSA and **C1**–**C4**, along with the approximate number of binding sites (*n*), the logarithmic form of the Stern–Volmer equation (Eq. 2) was used:

2 $$\mathit{log}\left(\frac{F_0-F}F\right)=nlogc_Q+logK_b$$

where *F*_*0*_ represents the initial fluorescence intensity of HSA, *F* represents the fluorescence intensity after the addition of a corresponding increment of the quencher (C1–C4), *c*_*Q*_ represents the quencher concentration in the sample, *n* represents the number of binding sites, and *K*_*b*_ is the binding constant. The values of *n* and *K*_*b*_ are determined from the slope and intercept of the logarithmic Stern–Volmer plot (Fig. 8, Fig. S4), respectively, and are listed in Table 2. As observed, the values of *n* for each derivative at each temperature are close to 1, implying that all of the studied compounds preferably bind to a single distinct binding site in the HSA molecule. The calculated binding constants were all found to be in order of 10^4^–10^6^ M^−1^, which is the optimal affinity range for binding of small molecules to HSA (Konkoľová et al. 2019), suggesting that compounds **C1**–**C4** could be effectively transported and distributed in the blood plasma in vivo. Furthermore, it is noteworthy that the binding constants calculated for derivatives **C1** and **C2** decreased at increasing temperatures, a finding which indicates a destabilization of the corresponding ligand–HSA complexes at higher temperatures. An opposite trend can be observed for the interaction of compounds **C3** and **C4** with HSA, suggesting that these two derivatives could be distributed more effectively in vivo, as they display the highest *K*_*b*_ values at 37 °C, the standard physiological temperature.

**Fig. 9:**
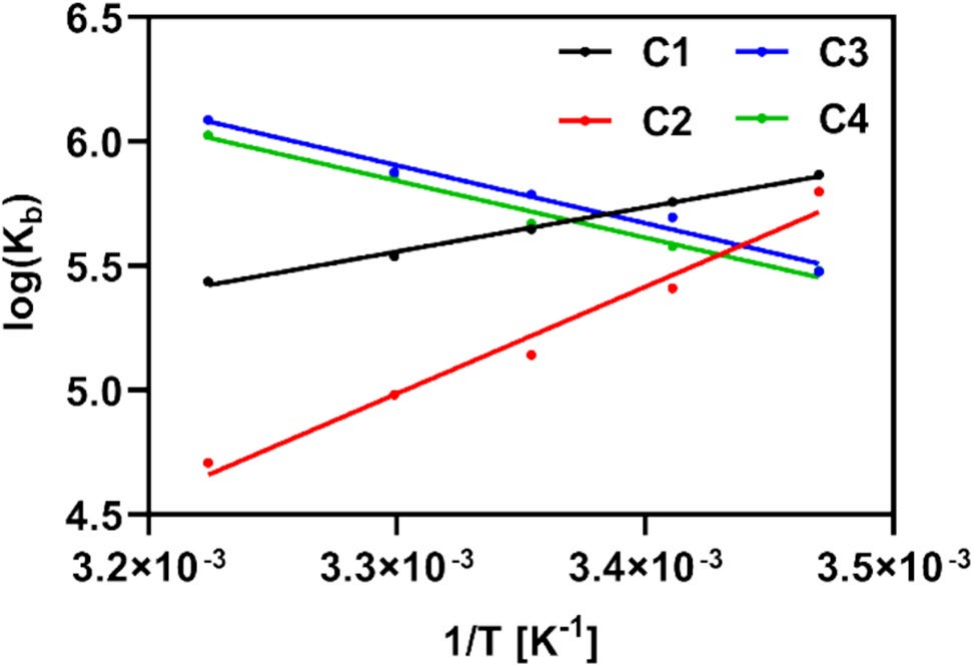
Van’t Hoff plot for the interaction of **C1**–**C4 (0**–**9.9** µM) with HSA (2.1 µM) at five different temperatures

The interaction between HSA and the derivatives **C1**–**C4** was studied further by calculating the thermodynamic parameters of the enthalpy change (ΔH) and the entropy change (ΔS). These values were determined using the Van’t Hoff equation (Eq. 3) and the slope and intercept of the corresponding Van’t Hoff plot (Fig. 9).

3 $$logK_b=-\frac{\Delta H}{2.303RT}+\frac{\Delta S}{2.303R}$$

where *K*_*b*_ is the binding constant, *T* is the thermodynamic temperature, *R* is the gas constant (R = 8.314 J.K^−1^.mol^−1^), and ΔH and ΔS represent the enthalpy and entropy changes, respectively. The Gibbs–Helmholtz equation (Eq. 4) was also applied to calculate the Gibbs free energy change (ΔG) for the individual reactions between HSA and **C1**–**C4** at each temperature point.

4 $$\Delta G=\Delta S-T\Delta S$$

where ΔG is the Gibbs free energy change, *T* is the thermodynamic temperature, and ΔH and ΔS represent the enthalpy and entropy changes, respectively. The calculated thermodynamic parameters are listed in Table 3.

**Table 3:** Thermodynamic parameters characterizing the interaction between HSA and compounds **C1**–**C4**

Compound	T (K)	ΔG (kJ.mol^−1^)	ΔH (kJ.mol^−1^)	ΔS (J.K^−1^.mol^−1^)	R^2^*
**C1**	288.15	− 32.37	− 33.94	− 55.90	0.9943
	293.15	− 31.93			
	298.15	− 32.23			
	303.15	− 32.15			
	310.15	− 32.29			
**C2**	288.15	− 31.99	−91.11	− 206.18	0.9715
	293.15	− 30.36			
	298.15	− 29.36			
	303.15	− 28.91			
	310.15	− 29.76			
**C3**	288.15	− 31.16	44.48	259.81	0.9786
	293.15	− 31.79			
	298.15	− 32.58			
	303.15	− 35.93			
	310.15	− 37.30			
**C4**	288.15	− 30.74	47.64	269.06	0.9830
	293.15	− 31.31			
	298.15	− 32.53			
	303.15	− 34.14			
	310.15	− 35.78			

*Correlation coefficient

In general, the values of ΔH and ΔS tend to provide a better insight into the primary binding forces involved in the interaction of small molecules with biomacromolecules. The negative values of ΔH and ΔS indicate the involvement of hydrogen bonding and van der Waals forces, while the positive ΔH and ΔS values are typical for hydrophobic interactions (Vilková et al. 2022). Positive ΔH values also suggest that the binding process is endothermic, and this is reflected in the higher *K*_*b*_ values at increasing temperatures. Conversely, exothermic binding is characterized by negative values of ΔH and decreasing *K*_*b*_ values at higher temperatures (Shahsavani et al. 2016). On this basis, it can be presumed that the binding of derivatives **C1** and **C2** to HSA is an enthalpy-driven exothermic process that is primarily mediated by hydrogen bonding and van der Waals forces. In contrast, compounds **C3** and **C4** seem to interact with HSA mainly through hydrophobic interactions via a process that is entropy driven. Lastly, the interactions between HSA and all of the studied compounds are characterized by negative ΔG values, which means that the binding processes can be considered spontaneous at each of the studied temperature points.

Based on the noticeable differences in the binding and thermodynamic parameters in the interactions of compounds **C1 **and** C2** and of compounds **C3** and **C4** with HSA, it can be concluded that the arrangement of -OH groups on the phenyl ring of the side chain plays a key role in the binding dynamics and orientation of the compounds in the binding site, despite the structures of all four compounds being very similar. Derivatives **C1** and **C2** are presumably oriented with their dihydroxyphenyl moiety close to the binding site, which allows the formation of hydrogen bonds through the -OH groups. On the other hand, arrangements of -OH groups in positions 2,5 (**C3**) or 2,4,6 (**C4**) seem to be unfavorable for such an interaction, and these compounds presumably interact with HSA through hydrophobic interactions mediated by the central coumarin moiety.

### Effect on HSA conformation

Synchronous fluorescence spectroscopy is a useful means of gaining basic insights into the conformational changes that occur in the proximity of HSA fluorophores upon the binding of small molecules. In this technique, both the emission and excitation monochromators are scanned simultaneously at a constant wavelength interval (Δλ). When the Δλ value is set at 15 nm, spectra which are characteristic of Tyr residues are observed, while at Δλ = 60 nm, spectra which are typical for Trp residues are recorded (Rudra et al. 2018). Similarly, as in the case of steady-state emission spectra, any shifts in the emission maxima of the synchronous spectra indicate a change in the polarity of the microenvironment surrounding the corresponding fluorophore (Chaves et al. 2018).

**Fig. 10:**
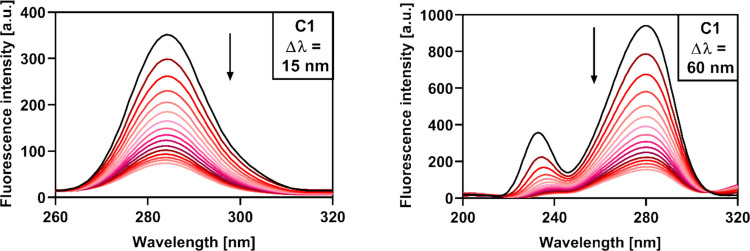
Synchronous fluorescence spectra of HSA (2.1 µM) in 10 mM PBS (pH 7.4) upon the addition of compound **C1** (0–29.1 µM)

As is shown in Fig. 10 and Fig. S5, the fluorescence of both Tyr and Trp residues was gradually quenched upon the addition of derivatives **C1**–**C4**. These findings correlate with the results obtained in the steady-state fluorescence experiments and indicate that HSA undergoes conformational changes as a result of **C1**–**C4** binding, a process which similarly affects both Tyr and Trp residues. As in the previous experiments, different effects of ligand binding on the microenvironment polarity were observed. Following the binding of **C1** and **C2** to HSA, no significant shifts of the emission maxima were observed in the synchronous spectra, implying that the fluorophore microenvironment polarity was not significantly affected. Interestingly, the binding of **C3** presumably caused conformational changes that led to an increase in polarity around Tyr residues and a decrease in polarity around the Trp residue, which corresponds to the results of the steady-state fluorescence experiments and supports the assumption that **C3** binds to HSA primarily through hydrophobic interactions. In the case of the trihydroxy derivative **C4**, the hydrophobic nature of the binding seems to be compensated by the presence of the extra -OH group, and therefore no significant shift of the emission maximum is observed in the Trp spectrum. Conformational changes accompanying the binding of this compound have, however, led to an increase in polarity around Tyr residues. Again, the results of the synchronous fluorescence experiments emphasize the considerable influence of the arrangement of the -OH groups on the spatial orientation of the ligands and their HSA-binding properties.

**Fig. 11:**
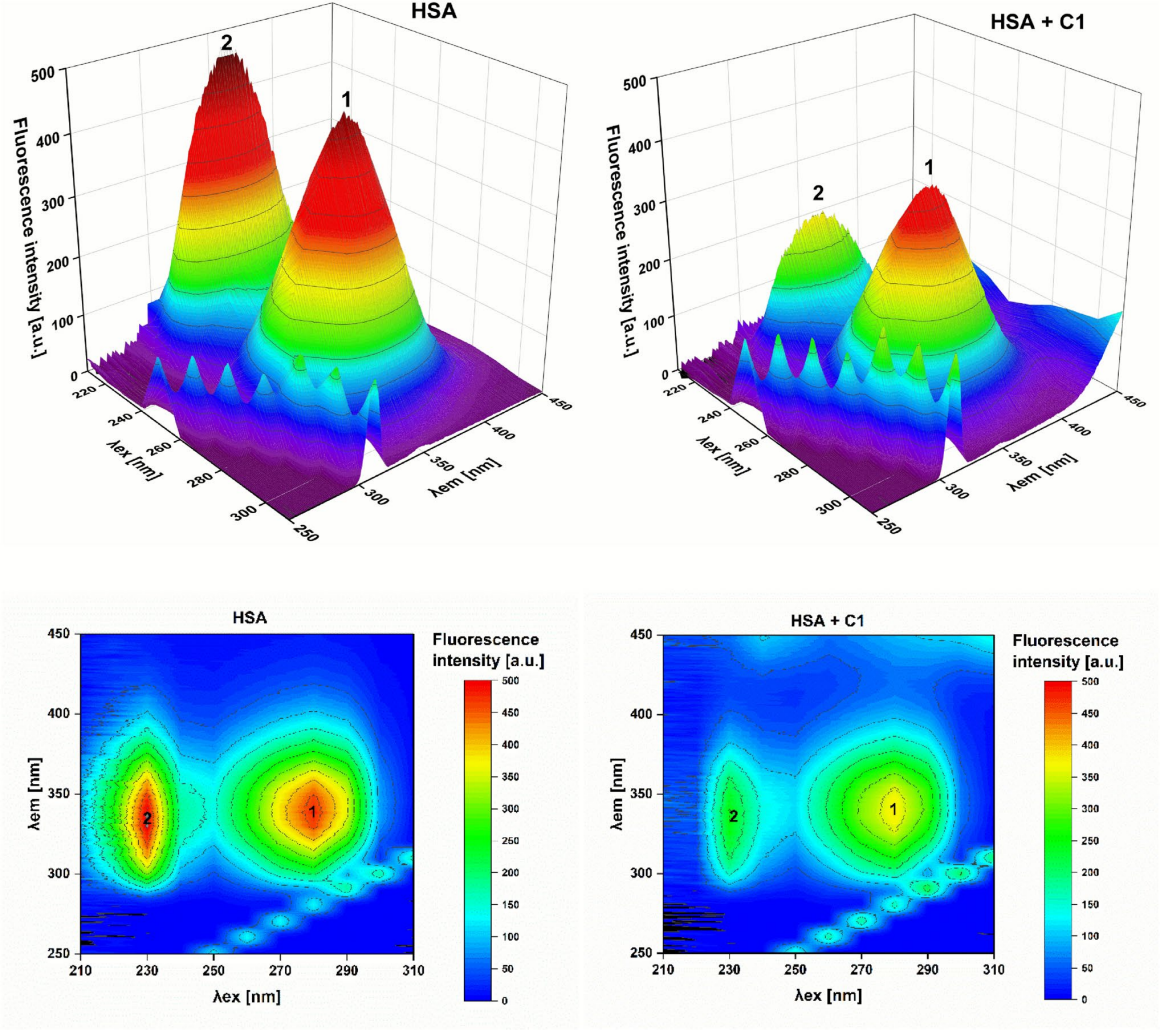
Three-dimensional fluorescence spectra (top) and the corresponding contour plots (bottom) of free HSA (2.6 µM) and HSA bound to **C1** (2.6 µM) in 10 mM PBS (pH 7.4)

Another useful method for studying the conformational changes of HSA upon binding to small molecules is three-dimensional fluorescence spectroscopy, a technique which not only offers information about the microenvironment of HSA fluorophores but also monitors structural changes occurring in the entire polypeptide backbone of the protein (Suo et al. 2018) and the associated protein surface area (Hashempour et al. 2020). The results of the 3D fluorescence spectra of free HSA (Fig. 11 and Figs. S6–S8, left) exhibit two major peaks labeled as peak 1 (λ_ex_ = 280 nm, λ_em_ = 340 nm) and peak 2 (λ_ex_ = 230 nm, λ_em_ = 335 nm). Changes in peak 1 are caused by π-π* transitions of HSA fluorophores and correspond to polarity alterations in the microenvironment around the Tyr and Trp residues, while peak 2 is the result of π-π* transitions of the C = O bond and reflects structural disturbances in the polypeptide backbone of HSA (Vilková et al. 2022). Upon the addition of derivatives **C1**–**C4**, the fluorescence intensities of both peaks were found to have decreased (Fig. 11 and Figs. S6–S8, right), but the decrease was far more profound in the case of peak 2, which confirms that the binding of **C1**–**C4** primarily affects the polypeptide backbone rather than the specific microenvironment of the corresponding fluorophores. In terms of the emission maxima, no significant wavelength shifts were observed in the case of peak 1. Concerning peak 2, red shifts of 5 nm, 4 nm, and 5 nm were observed after the binding of compounds **C1**, **C2**, and **C4**, respectively, while a blue shift of 6 nm was induced by the binding of derivative **C3**. These results correlate with the observations obtained from the steady-state fluorescence spectra.

### Determination of the preferred HSA binding site

**Fig. 12:**
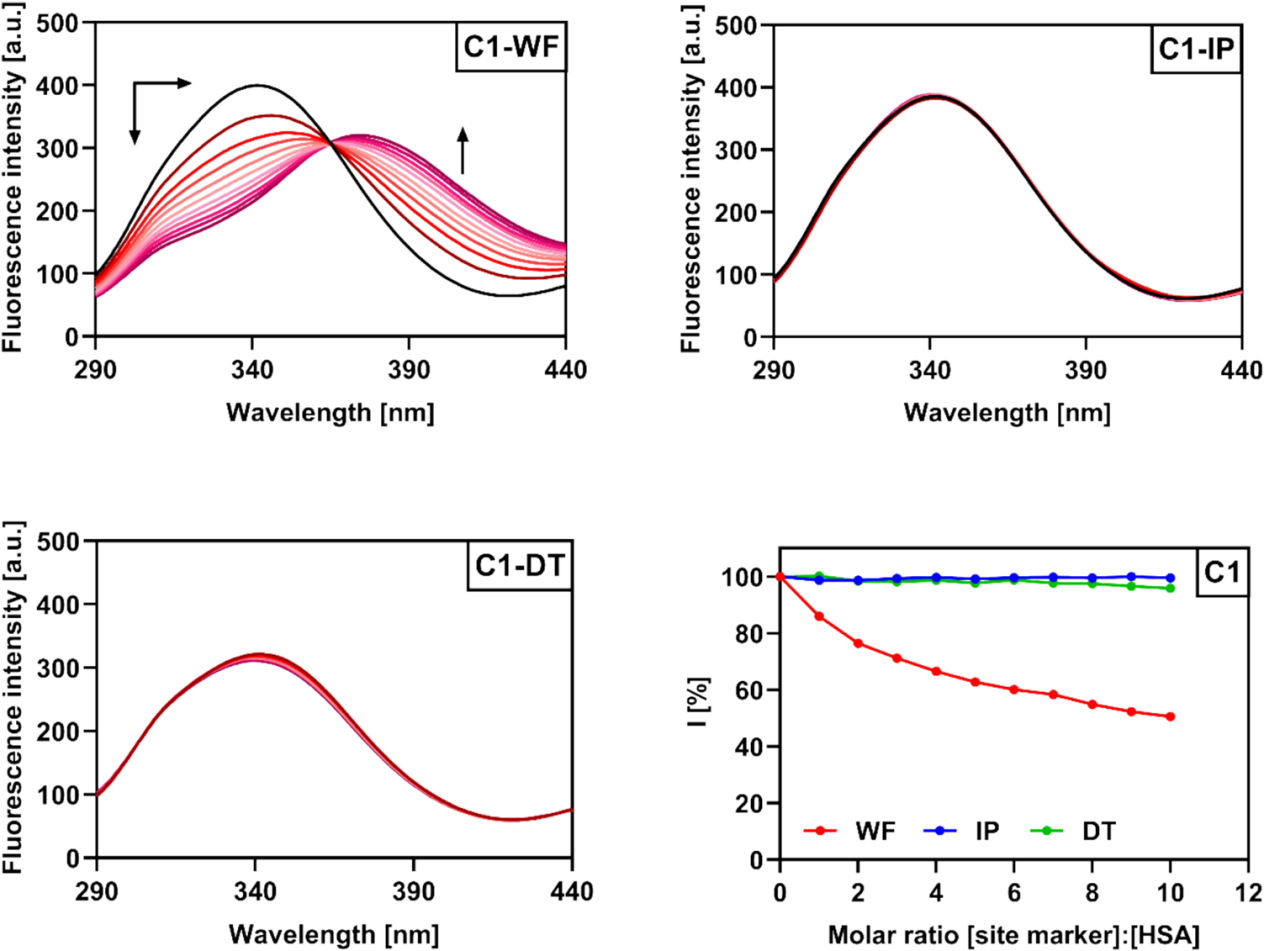
Emission spectra of HSA-**C1** (1:1) complexes in 10 mM PBS (pH 7.4) upon addition of site markers warfarin (WF), ibuprofen (IP), and digitoxin (DT) along with a graphical analysis of the competitive experiment. Molar ratios of HSA-**C1**/site markers were as follows: 1:1, 1:2, 1:3, 1:4, 1:5, 1:6, 1:7, 1:8, 1:9, and 1:10

The results of the previous calculations have proven that all of the studied derivatives **C1**–**C4** occupy at least one binding site in the HSA structure, but in order to determine the preferred binding site, competitive displacement assays were performed using warfarin (WF), ibuprofen (IP), and digitoxin (DT) as standard markers of sites I, II, and III, respectively (Fan et al. 2024; Vilková et al. 2022). The HSA-coumarin (1:1) complexes were excited and gradually titrated with equimolar increments of the respective site marker (Fig. 12, Figs. S9–S11). In the case of warfarin, fluorescence quenching of the HSA-coumarin complexes was observed initially and new emission maxima gradually formed at around 375 nm. No significant changes in the emission spectra were observed after the addition of IP and DT, indicating that derivatives **C1**–**C4** primarily occupy Sudlow site I, where they were gradually replaced by WF. WF is also a coumarin derivative, and this would suggest that compounds **C1**–**C4** would be likely to bind at the same site. However, the calculated number of binding sites (1.01–1.17) indicates that Sudlow site I may not be the sole binding site of the studied compounds; it is also possible that they can fractionally bind to sites II and III.

The results were analyzed using Eq. (5) and the corresponding graphs (Fig. 12, Figs. S9–S11, bottom right)

5 $$I={(F}_0/F)\times100\%$$

where *I* represents the percentage of the initial fluorescence intensity (F_0_) and *F* is the fluorescence intensity after the addition of the corresponding site marker.

**Fig. 13:**
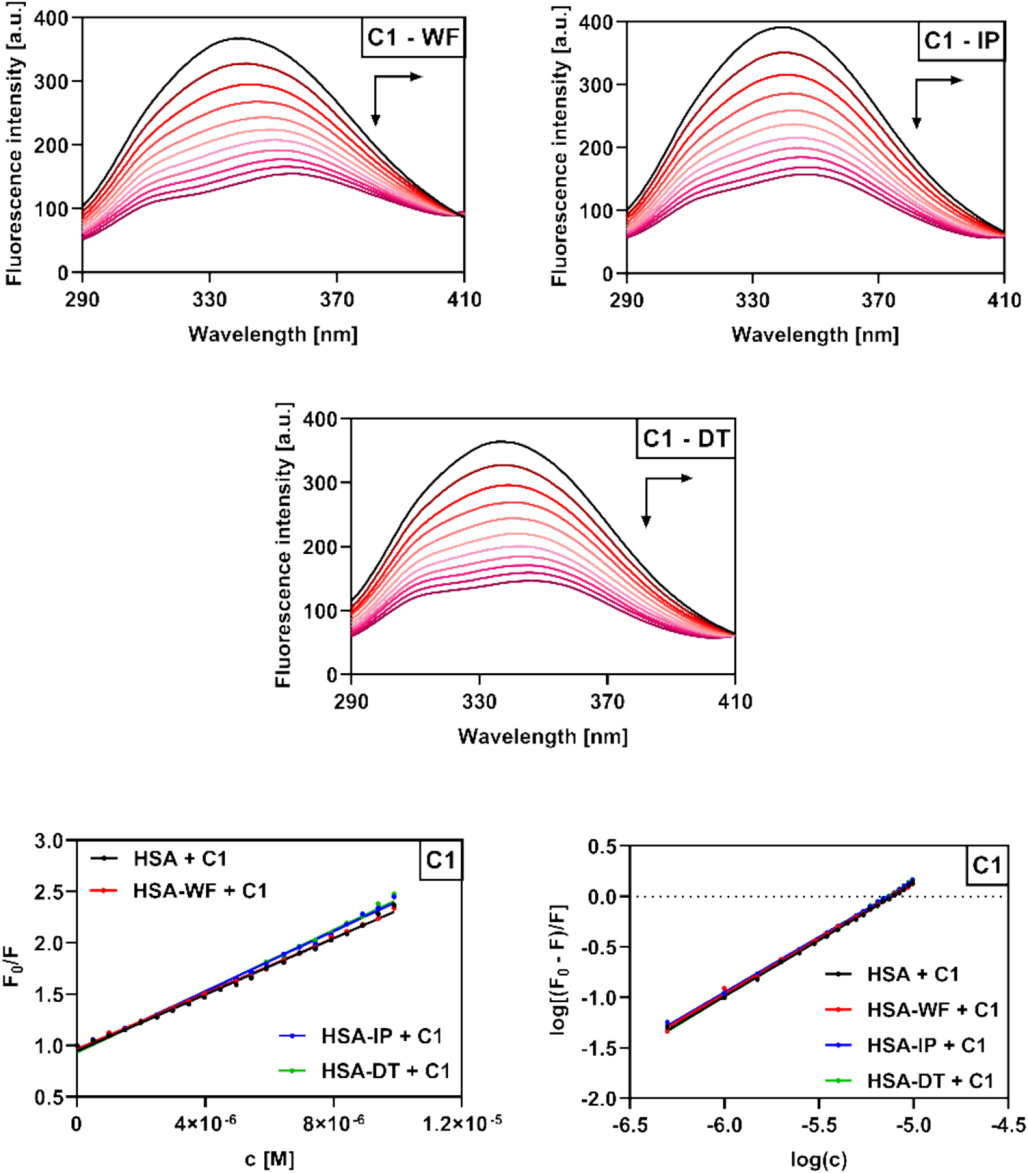
Emission spectra of HSA-site marker (1:1) complexes in 10 mM PBS (pH 7.4) upon addition of **C1** (0–9.9 µM) along with the corresponding Stern–Volmer plot (bottom left) and logarithmic Stern–Volmer plot (bottom right)

In order to gain a fuller understanding of the preferred binding site, reverse experiments were performed in which the HSA-site marker (1:1) complexes were gradually titrated with equimolar increments of compounds **C1**–**C4**. Previous studies have suggested that if a compound preferably binds to the same site as the corresponding marker, the *K*_*b*_ value of the interaction is lower than the *K*_*b*_ value of its binding to free HSA since there is no need for the ligand to compete with the site marker (Bi et al. 2009). The addition of the studied derivatives to the HSA-marker complexes resulted in fluorescence quenching accompanied by red (**C1**, **C2**, **C4**) or blue (**C3**) shifts (Fig. 13, Figs. S12–S14), a finding which corroborates the results of the free HSA titration assays (Fig. 7, Figs. S1–S3). The results were analyzed identically using linear and logarithmic Stern–Volmer plots (Fig. 13, Fig. S15) and corresponding Eqs. (1) and (2).

Table 4 shows the calculated *K*_*SV*_ and *K*_*b*_ values for the individual tested systems. The results clearly show that the *K*_*SV*_ values for the coumarin-HSA complexes either increased or decreased in the presence of WF, IP, and DT, a shift which could be attributed to interactions between the studied derivatives and individual site markers (Tu et al. 2015). More significantly, however, the *K*_*b*_ values of compounds **C1**–**C4** when bound to the HSA-WF complex were found to be lower than those observed in the case of free HSA binding. Conversely, the binding constants either increased or remained almost the same in the presence of ibuprofen and digitoxin. These findings further support our assumption that compounds **C1**–**C4** compete with warfarin for binding at Sudlow site I.

**Table 4:** Stern–Volmer quenching constants (*K*_*SV*_) and binding constants (*K*_*b*_*)* describing the interactions between compounds **C1**–**C4** and individual HSA-site marker (1:1) complexes

System	HSA		HSA-WF		HSA-IP			HSA-DT	
Comp. ↓	***K***_***SV***_** (× 10**^**5**^** M**^**−1**^**)**	***K***_***b***_** (× 10**^**5**^** M**^**−1**^**)**	***K***_***SV***_** (× 10**^**5**^** M**^**−1**^**)**	***K***_***b***_** (× 10**^**5**^** M**^**−1**^**)**	***K***_***SV***_** (× 10**^**5**^** M**^**−1**^**)**	***K***_***b***_** (× 10**^**5**^** M**^**−1**^**)**		***K***_***SV***_** (× 10**^**5**^** M**^**−1**^**)**	***K***_***b***_** (× 10**^**5**^** M**^**−1**^**)**
C1	1.16 ± 0.13	4.44 ± 0.17	1.31 ± 0.06	3.97 ± 0.13	1.39 ± 0.04		7.28 ± 0.07	1.40 ± 0.03	5.95 ± 0.11
C2	0.91 ± 0.12	1.39 ± 0.10	1.03 ± 0.06	0.89 ± 0.06	1.11 ± 0.02		8.10 ± 0.05	1.02 ± 0.06	2.28 ± 0.03
C3	1.29 ± 0.10	5.11 ± 0.10	1.66 ± 0.12	2.88 ± 0.18	1.36 ± 0.03		5.68 ± 0.11	1.26 ± 0.08	5.50 ± 0.05
C4	1.69 ± 0.03	4.99 ± 0.15	1.30 ± 0.09	2.78 ± 0.11	1.36 ± 0.12		9.41 ± 0.22	1.38 ± 0.14	8.15 ± 0.16

### Molecular modelling and docking simulations

As had been predicted using the Molsoft tool (MolSoft 2025), the tested molecules were found to be in a neutral form at pH 7.4. Using this as a framework, the structures of the tested compounds were built and modelled and then used for docking to the human Topo I-DNA complex (PDB ID: 1T8I) (Staker et al. 2005a, b) and HSA (PDB ID: 1AO6) (Sugio et al. 1998, 1999). The first docking simulation was performed to predict possible interaction modes of the derivatives and the hTopoI-DNA complex, and the second to identify a possible binding site on HSA.

**Fig. 14:**
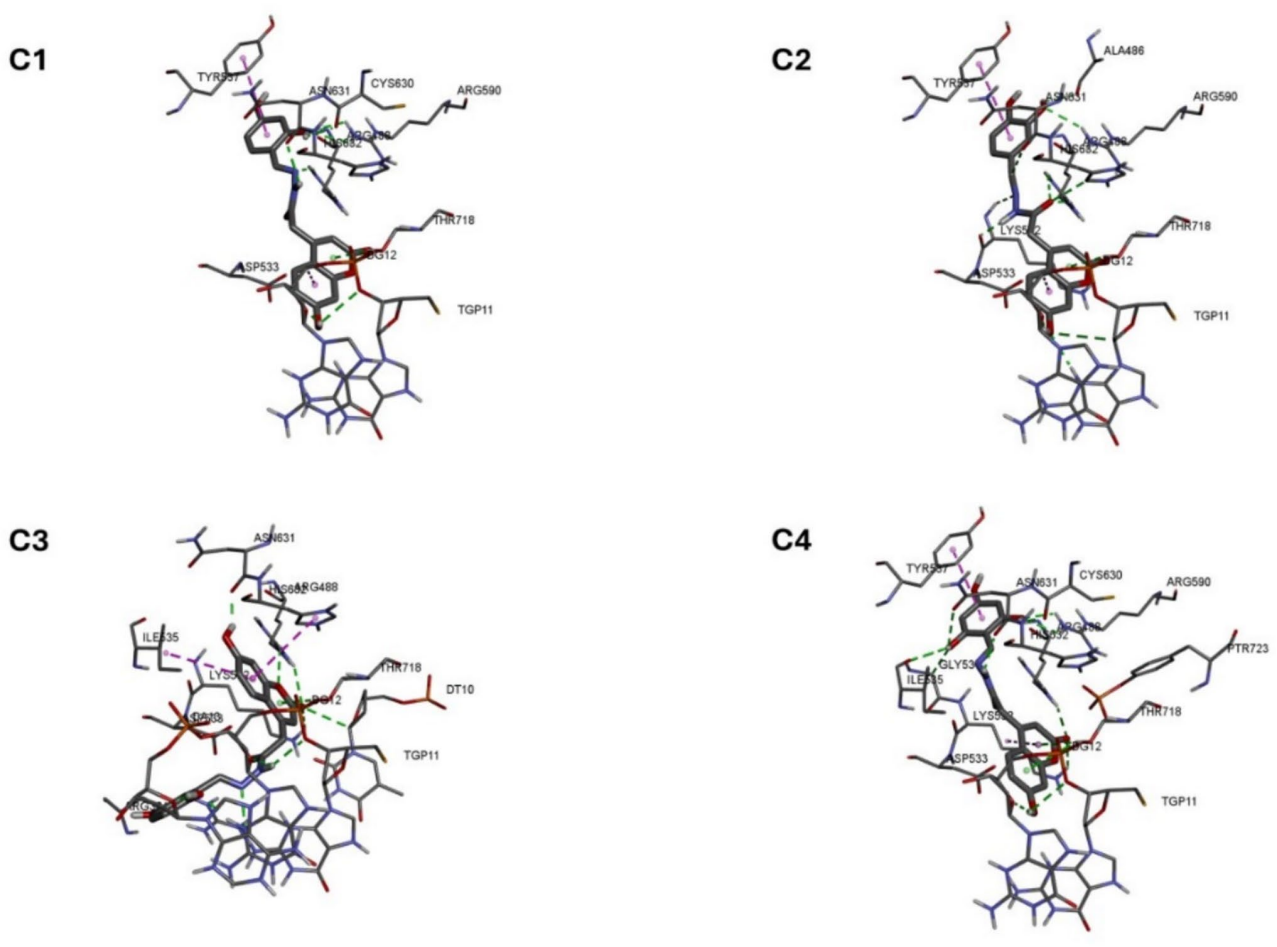
Predicted binding poses for molecules of compounds **C1**–**C4** within the hTopo I-DNA complex (PDB ID: 1T8I (Staker et al. 2005a, b)). Interactions: hydrogen bonds (green dashed line) and hydrophobic bonds (pink dashed line)

The predicted binding poses for molecules of compounds **C1**–**C4** within the hTopo I-DNA complex are presented in Fig. 14. As can be seen, the phenyl rings of the side chains of molecules **C1**, **C2**, and **C4** are oriented similarly, while in **C3** they are oriented differently (Fig. 14). Interactions between substituent atoms and/or linker atoms (between the coumarin ring and the benzene ring) and the amino acid residues of the protein play an important role in the binding of the tested molecules. The poses of **C1** and **C4** are mainly stabilized by hydrogen bonds formed between substituents in the phenyl moiety (in position 2 and in positions 2 and 6, respectively) and the amino acid residues, while **C2** is mainly stabilized by hydrogen bonds between atoms in the linker -C–CO-NH-N = C- and the amino acid residues. There are no significant differences in predicted binding free energy values for **C1**–**C4** (Table S1).

**Fig. 15:**
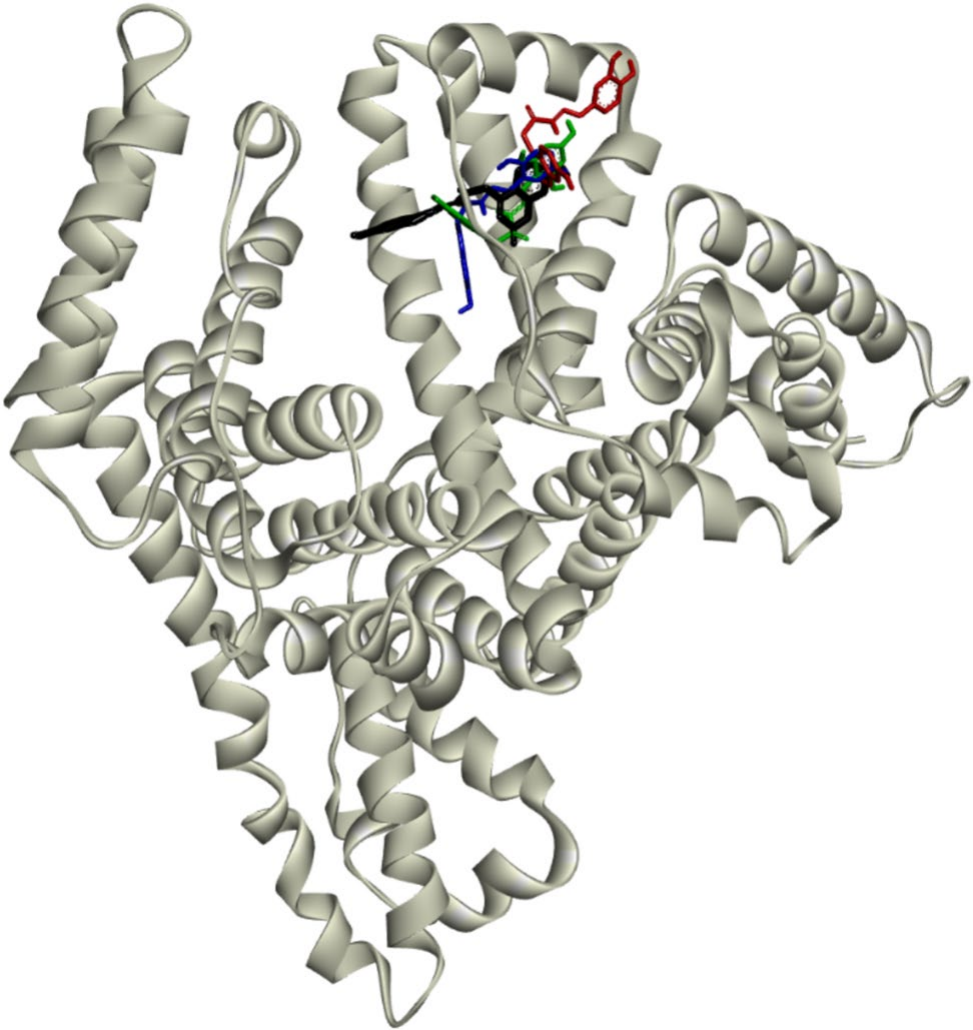
Graphical representation of the lowest energy conformation complex of compounds **C1**–**C4** with HSA (PDB ID: 1AO6 (Sugio et al. 1998, 1999)). The protein is expressed using a ribbon model (grey) and the molecules of the tested compounds are expressed in the form of rods: **C1** (black), **C2** (red), **C3** (blue), and **C4** (green)

The HSA complex with the lowest energy conformation of the molecules of the tested compounds is presented in Fig. 15. As can be seen, all of the tested molecules are located in binding site III of the IB subdomain of HSA. The amino acid residues involved in these interactions are listed in Table S2. The interactions between these residues and the molecules of the tested compounds are mediated by van der Waals interactions, hydrogen bonding, and hydrophobic interactions. The latter play a more significant role in the binding of **C3** and **C4** than in the other derivatives, a finding that is in good agreement with the experiment-based predictions (see Sect. "Interaction with HSA").

The predicted binding free energy values (Table 5) suggest that derivative **C1** forms the strongest bind to HSA, while the binding of **C2** is deemed to be the weakest. The binding free energies predicted by the computer modelling studies (Table 5) are generally slightly higher than those obtained from the experimental results, a minor discrepancy which may be a consequence of the empirical scoring function used during the docking calculations (Morris et al. 1998) and/or the omission of microenvironment effects in the computational studies (Morris et al. 1998; Pantsar and Poso 2018). These factors might also account for the contradictions between the experimental and computational results concerning the primary binding site of derivatives **C1**–**C4**. The difference between the experimental identification of Sudlow site I and the docking-predicted preference for site III can likely be explained by the distinct physical assumptions of the two methods. Blind docking relies on a static crystal structure and an empirical scoring function that is unable to integrate the full scope of factors such as solvent effects, buffer ions, and entropic contributions. Under these essentially simplified conditions, ligands are often predicted to occupy compact hydrophobic pockets such as site III, where the calculated interaction energy appears slightly more favorable (Morris et al. 1998; Pantsar and Poso 2018; Zsila 2013). In contrast, fluorescence-based displacement assays are performed in PBS, which preserves the native ionic environment and allows the intrinsic domain mobility of HSA (Hashempour et al. 2020; Suo et al. 2018). Subdomain IIA, containing Sudlow site I, is known for its greater degree of conformational flexibility, thereby enabling induced-fit adjustments that promote ligand accommodation (Yue et al. 2018); it is precisely this type of effect that cannot be effectively determined using docking simulations. Additionally, the binding of ligands at site I is associated with significant entropic gains linked to water release and side-chain rearrangements (Vilková et al. 2022), effects which are not reflected in the docking score but which contribute substantially to the experimentally observed affinity. These considerations indicate that site I is the dominant binding region under physiological-like conditions, whereas site III likely represents a minor, energetically favorable alternative primarily detected in silico.

**Table 5:** Predicted free energies of binding for the tested compounds with HSA (PDB ID: 1AO6 (Sugio et al. 1998, 1999))

**Compound**	**Free energy of binding**^a^	
	(kcal.mol^−1^)	(kJ.mol^−1^)
**C1**	− 5.63	− 23.57
**C2**	− 5.04	− 21.10
**C3**	− 5.30	− 22.19
**C4**	− 5.31	− 22.23

^a^For T = 293.15 K

## Conclusion

This study provides a comprehensive evaluation of four newly synthesized 4-substituted 7-hydroxycoumarins, revealing a clear multitarget profile relevant to anticancer drug development. Derivatives **C1**–**C4** demonstrated marked biological activity, perhaps the most significant of which is the selective cytotoxicity toward A549 lung carcinoma cells while sparing normal fibroblasts and the concentration-dependent inhibition of Topo I, with **C1** emerging as the most active compound in this respect. The results of the fluorescence spectroscopy assays revealed strong binding to HSA with binding constants in the optimal affinity range of 10^4^–10^6^ M^−1^. Displacement assays confirmed Sudlow site I as the primary binding site, whereas molecular docking suggested an alternative preference for site III, pointing to the possibility of minor multisite interactions. The binding of the ligands induced conformational adjustments in the protein which are consistent with the partial unfolding of the HSA domains and the stabilization of the complex. Overall, these findings strongly suggest that derivatives **C1**–**C4** are promising coumarin-based scaffolds that combine selective anticancer activity with favorable pharmacokinetic characteristics, indicating their potential for further optimization in the development of new anticancer drugs.

## Supplementary Information

Below is the link to the electronic supplementary material.

ESM1 (PDF 2.15 MB)

## Data Availability

The data collected as part of the research for this article are not publicly available due to the fact that they have not yet been uploaded to any public repository. However, the data is accessible through the corresponding author upon reasonable request.
