# Supplementary material for: Multitarget evaluation of 4-substituted 7-hydroxycoumarin derivatives: anticancer activity, topoisomerase I inhibition, and interaction with human serum albumin
Source: Naunyn Schmiedebergs Arch Pharmacol. 2026 Feb 12;399(7):10873–92. doi: 10.1007/s00210-026-05062-w (PMC13152983; doi:10.1007/s00210-026-05062-w)
Supplement: Supplementary file 1 — (PDF 2.15 MB) [file 210_2026_5062_MOESM1_ESM.pdf]

Multitarget Evaluation of 4-Substituted 7-Hydroxycoumarin Derivatives: Anticancer Activity, Topoisomerase I Inhibition and Interaction with Human Serum Albumin

Adrián Gucký[a], Martin Majerník[b], Slávka Hamuľaková[c], Katarzyna E. Nowak[d], Rastislav Jendželovský[b], Peter Fedoročko[b], Mária Kožurková[a*]

[a]Department of Biochemistry, Institute of Chemistry, Faculty of Science, P. J. Šafárik University in Košice, Moyzesova 11, 040 01, Košice, Slovak Republic

[b]Department of Cell Biology, Institute of Biology and Ecology, Faculty of Science, P. J. Šafárik University in Košice, Šrobárova 2, 040 01, Košice, Slovak Republic

[c]Department of Organic Chemistry, Institute of Chemistry, Faculty of Science, P. J. Šafárik University in Košice, Moyzesova 11, 040 01, Košice, Slovak Republic

[d]Department of Oncobiology and Epigenetics, Faculty of Biology and Environmental Protection, University of Lodz, Pomorska 141/143, 90-236 Lodz, Poland

*Corresponding author. Telephone number: +421 55 234 2663 E-mail address: maria.kozurkova@upjs.sk

SUPPLEMENTARY INFORMATION

Contents

Fig. S1 Emission spectra of HSA (2.1 μM) in 10 mM PBS (pH 7.4) upon addition of **C2** (0-9.9 μM) at five different temperatures

Fig. S2 Emission spectra of HSA (2.1 μM) in 10 mM PBS (pH 7.4) upon addition of **C3** (0-9.9 μM) at five different temperatures

Fig. S3 Emission spectra of HSA (2.1 μM) in 10 mM PBS (pH 7.4) upon addition of **C4** (0-9.9 μM) at five different temperatures

Fig. S4 Stern-Volmer plots (left) and logarithmic Stern-Volmer plots (right) for the fluorescence quenching of HSA (2.1 μM) by compounds **C2**-**C4** (0-9.9 μM) at five different temperatures

Fig. S5 Synchronous fluorescence spectra of HSA (2.1 μM) in 10 mM PBS (pH 7.4) upon addition of compounds **C2**-**C4** (0-29.1 μM)

Fig. S6 Three-dimensional fluorescence spectra (top) and the corresponding contour plots (bottom) of free HSA (2.6 μM) and HSA bound to **C2** (2.6 μM) in 10 mM PBS (pH 7.4)

Fig. S7 Three-dimensional fluorescence spectra (top) and the corresponding contour plots (bottom) of free HSA (2.6 μM) and HSA bound to **C3** (2.6 μM) in 10 mM PBS (pH 7.4)

Fig. S8 Three-dimensional fluorescence spectra (top) and the corresponding contour plots (bottom) of free HSA (2.6 μM) and HSA bound to **C4** (2.6 μM) in 10 mM PBS (pH 7.4)

Fig. S9 Emission spectra of HSA-**C2** (1:1) complexes in 10 mM PBS (pH 7.4) upon addition of site markers warfarin (WF), ibuprofen (IP) and digitoxin (DT) along with a graphical analysis of the competitive experiment. Molar ratio of HSA-**C2**:site marker was 1:1, 1:2, 1:3, 1:4, 1:5, 1:6, 1:7, 1:8, 1:9 and 1:10

Fig. S10 Emission spectra of HSA-**C3** (1:1) complexes in 10 mM PBS (pH 7.4) upon addition of site markers warfarin (WF), ibuprofen (IP) and digitoxin (DT) along with a graphical analysis of the competitive experiment. Molar ratio of HSA-**C3**:site marker was 1:1, 1:2, 1:3, 1:4, 1:5, 1:6, 1:7, 1:8, 1:9 and 1:10

Fig. S11 Emission spectra of HSA-**C4** (1:1) complexes in 10 mM PBS (pH 7.4) upon addition of site markers warfarin (WF), ibuprofen (IP) and digitoxin (DT) along with a graphical analysis of the competitive experiment. Molar ratio of HSA-**C4**:site marker was 1:1, 1:2, 1:3, 1:4, 1:5, 1:6, 1:7, 1:8, 1:9 and 1:10

Fig. S12 Emission spectra of HSA-site marker (1:1) complexes in 10 mM PBS (pH 7.4) upon addition of **C2** (0-9.9 μM)

Fig. S13 Emission spectra of HSA-site marker (1:1) complexes in 10 mM PBS (pH 7.4) upon addition of **C3** (0-9.9 μM)

Fig. S14 Emission spectra of HSA-site marker (1:1) complexes in 10 mM PBS (pH 7.4) upon addition of **C4** (0-9.9 μM)

Fig. S15 Stern-Volmer plots (left) and logarithmic Stern-Volmer plots (right) for the fluorescence quenching of HSA-site marker (1:1) complexes by compounds **C2**-**C4** (0-9.9 μM).

Table S1 Predicted free energies of binding for the tested compounds with the hTopo I-DNA complex (PDB ID: 1T8I)

Table S2 Amino acid residues involved in the interactions between the tested compounds and HSA (PDB ID: 1AO6)

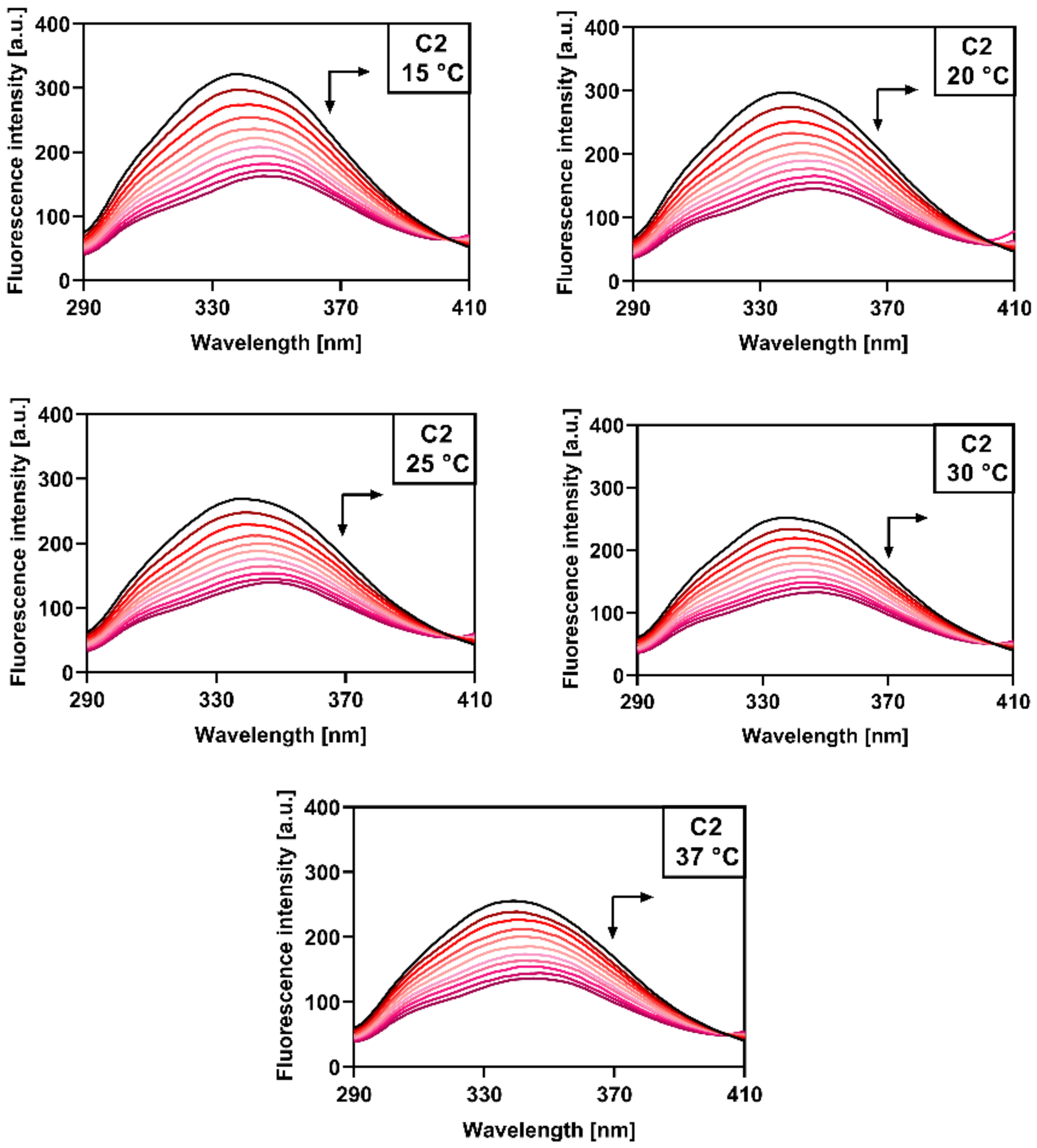

Fig. S1 Emission spectra of HSA (2.1 μM) in 10 mM PBS (pH 7.4) upon addition of **C2** (0-9.9 μM) at five different temperatures

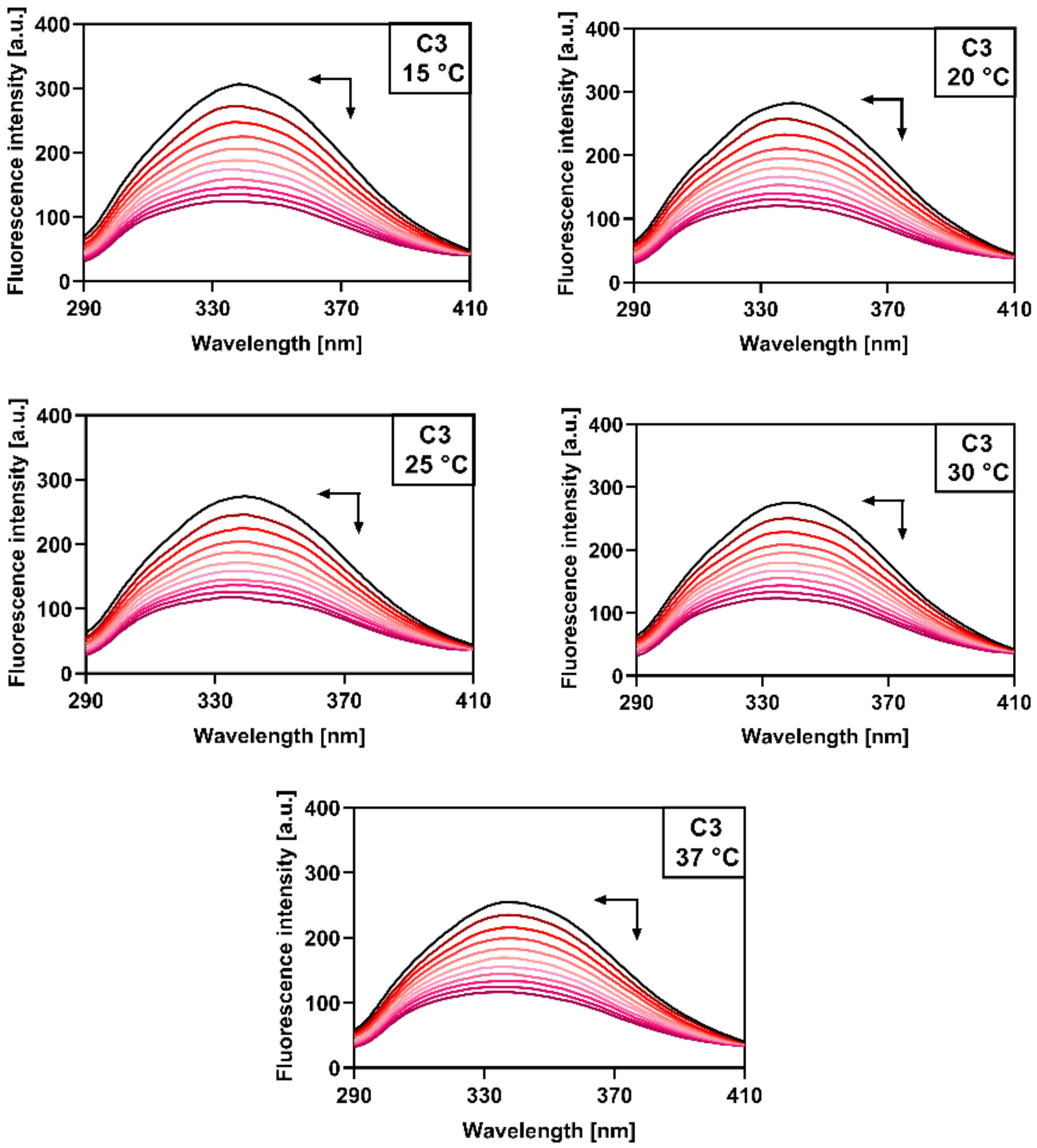

Fig. S2 Emission spectra of HSA (2.1 μM) in 10 mM PBS (pH 7.4) upon addition of **C3** (0-9.9 μM) at five different temperatures

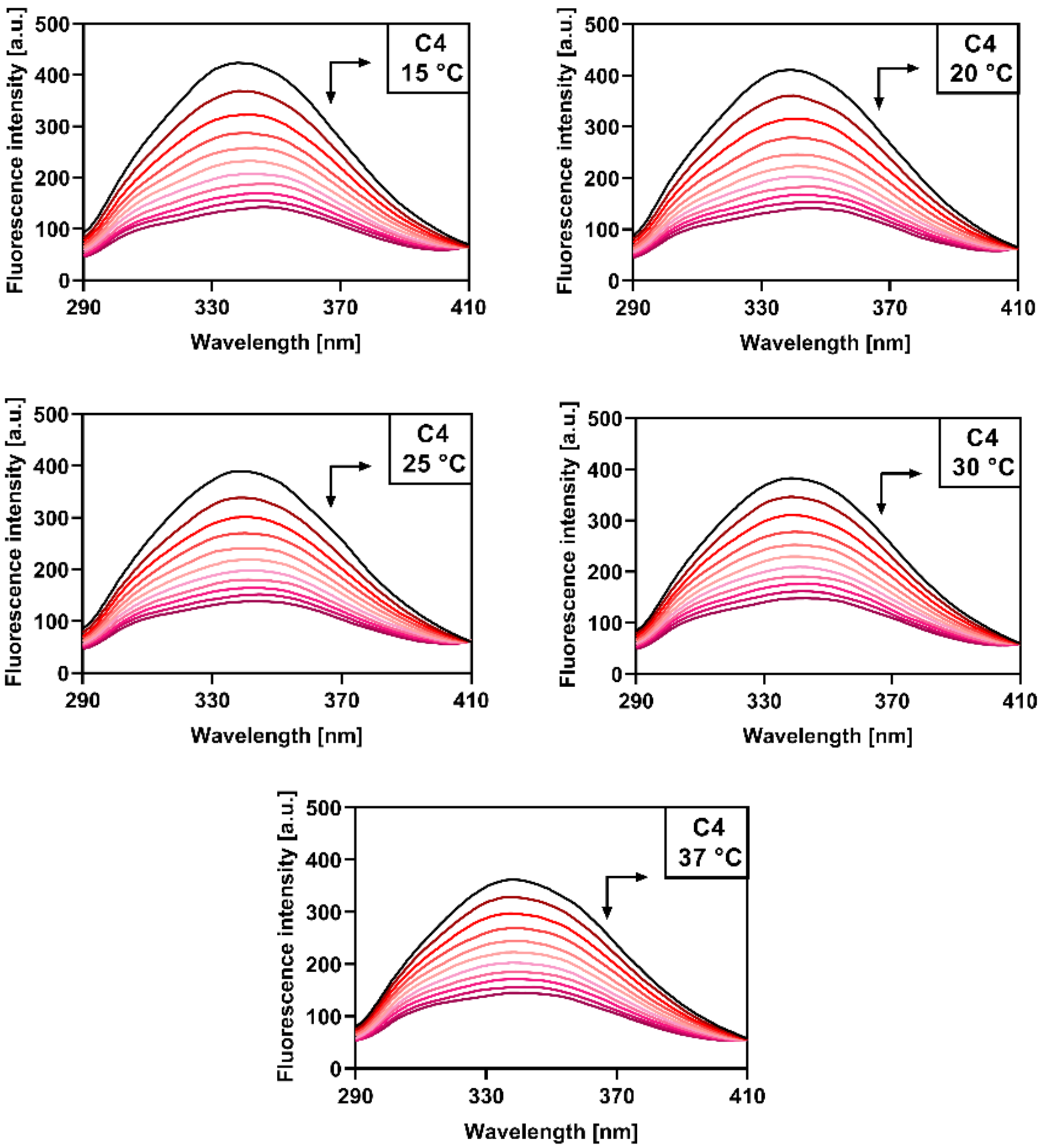

Fig. S3 Emission spectra of HSA (2.1 μM) in 10 mM PBS (pH 7.4) upon addition of **C4** (0-9.9 μM) at five different temperatures

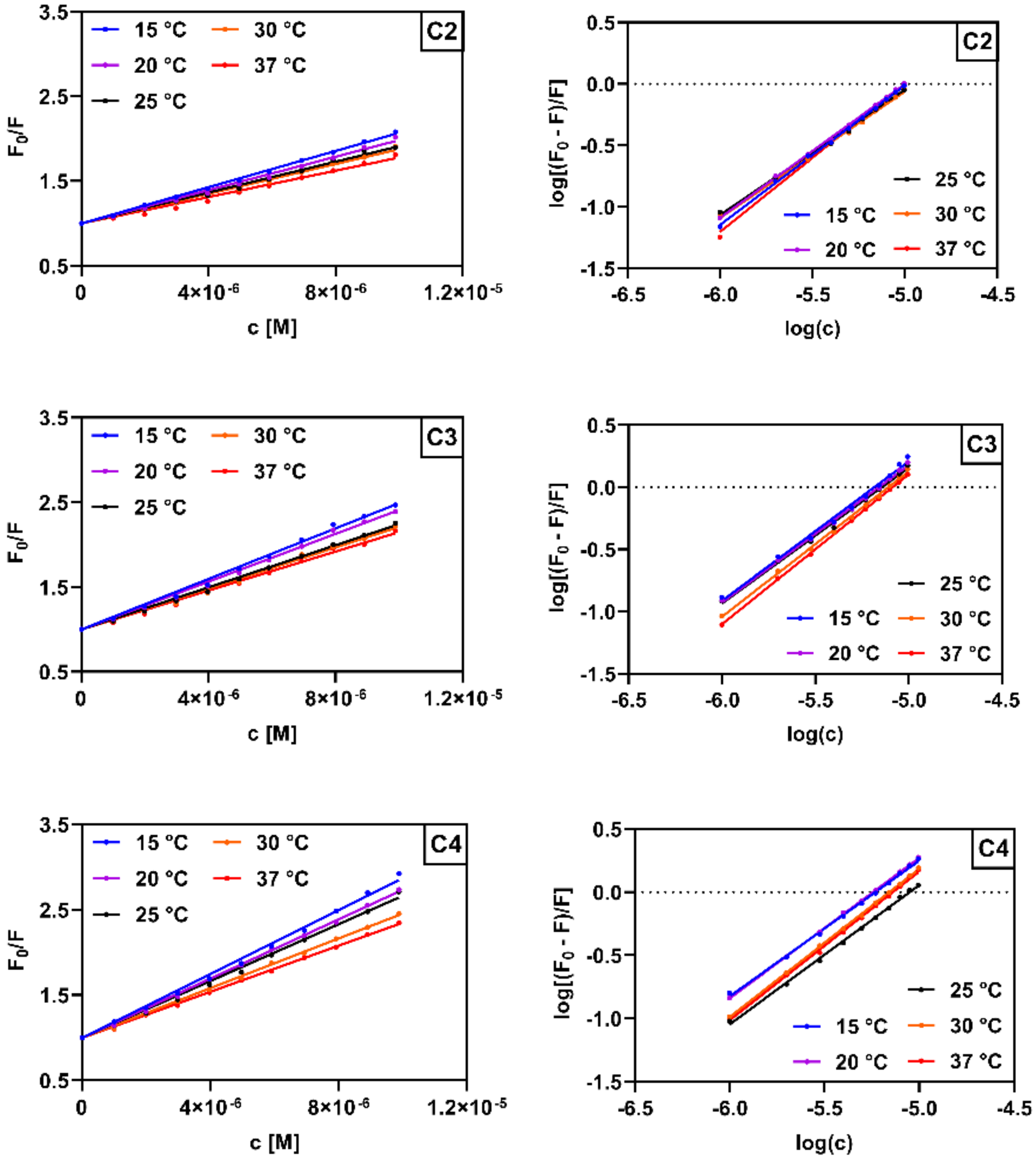

Fig. S4 Stern-Volmer plots (left) and logarithmic Stern-Volmer plots (right) for the fluorescence quenching of HSA (2.1 µM) by compounds **C2**-**C4** (0-9.9 µM) at five different temperatures

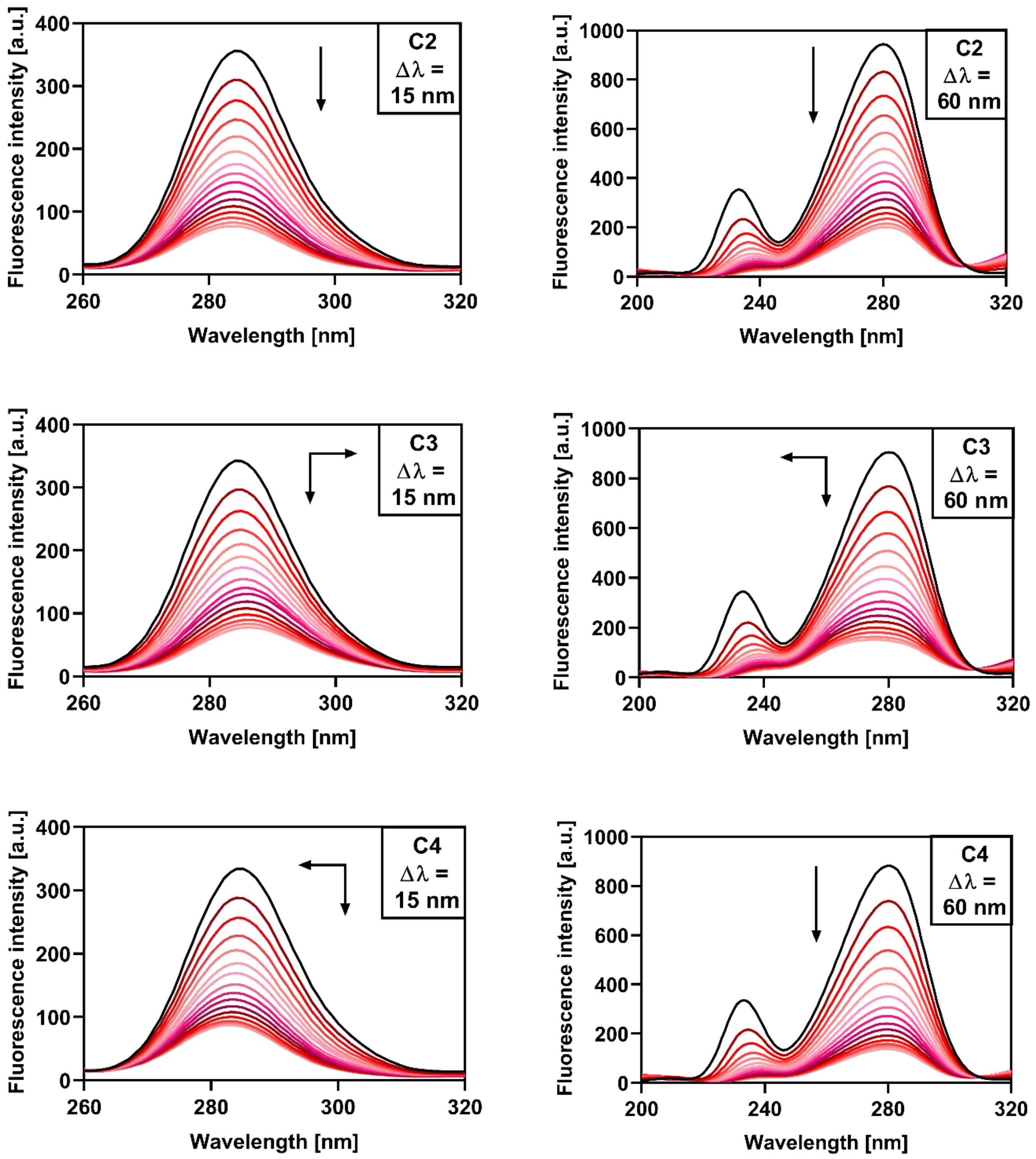

Fig. S5 Synchronous fluorescence spectra of HSA (2.1 µM) in 10 mM PBS (pH 7.4) upon addition of compounds **C2**-**C4** (0-29.1 µM)

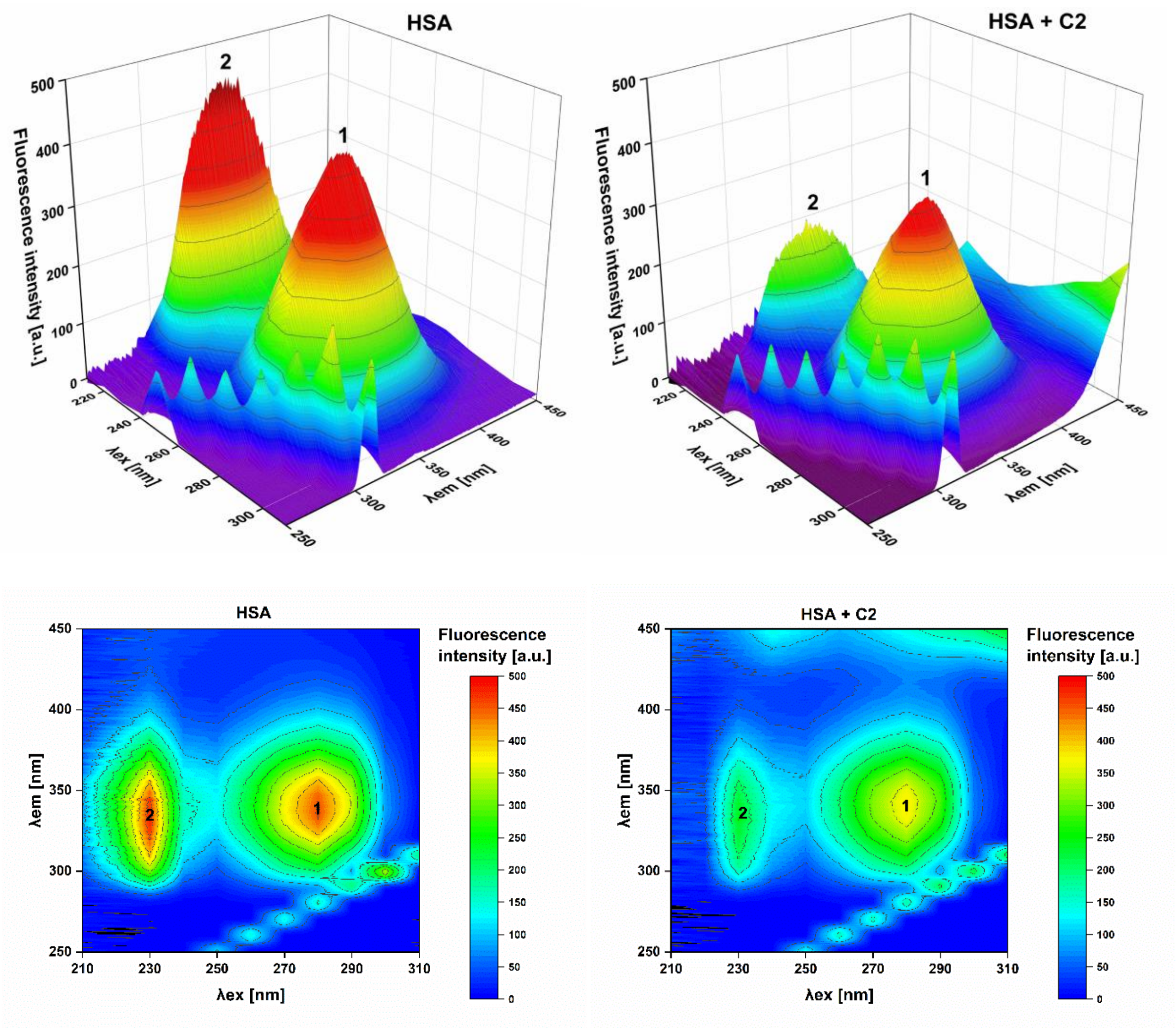

Fig. S6 Three-dimensional fluorescence spectra (top) and the corresponding contour plots (bottom) of free HSA (2.6 µM) and HSA bound to **C2** (2.6 µM) in 10 mM PBS (pH 7.4)

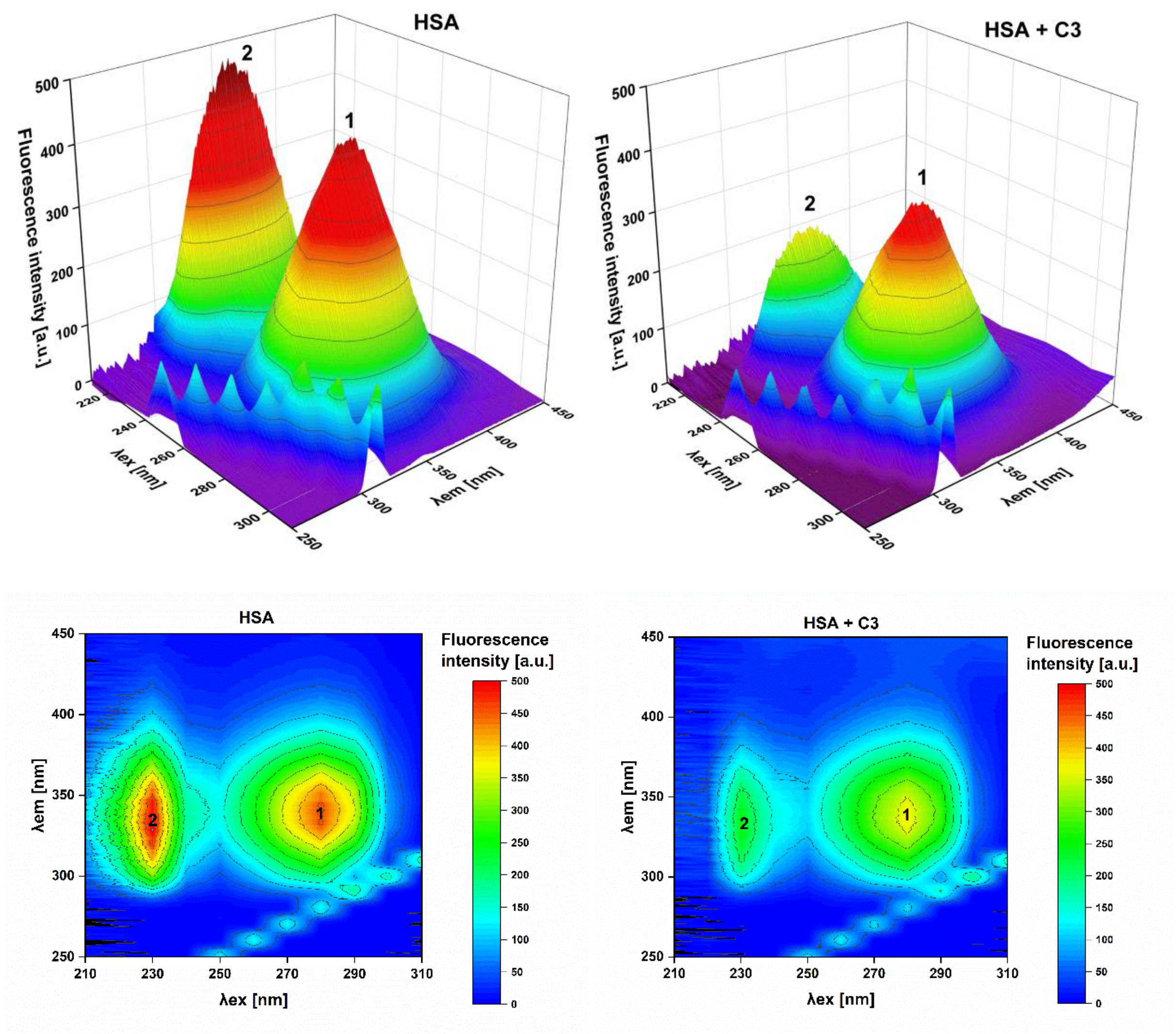

Fig- S7 Three-dimensional fluorescence spectra (top) and the corresponding contour plots (bottom) of free HSA (2.6 μM) and HSA bound to **C3** (2.6 μM) in 10 mM PBS (pH 7.4)

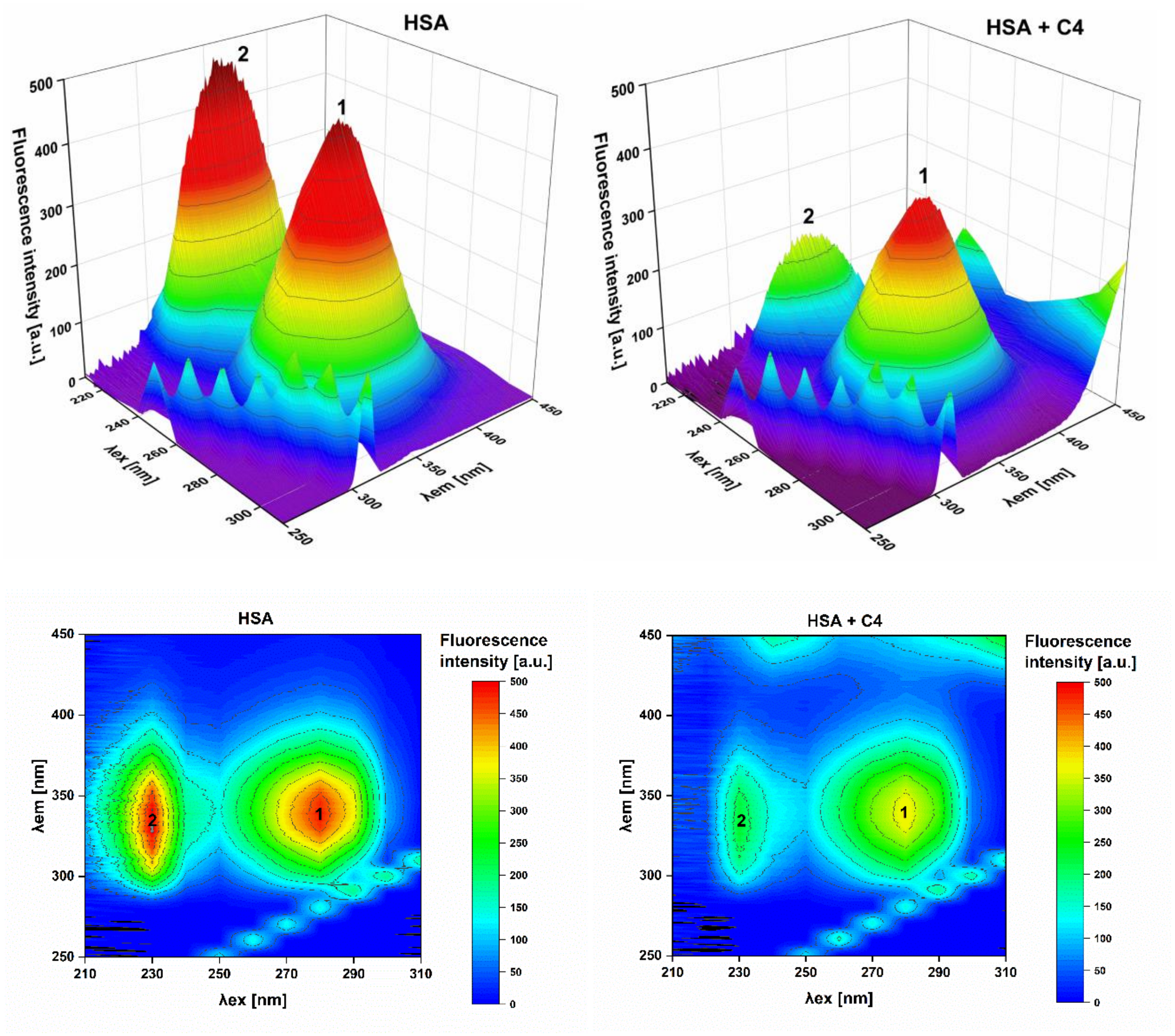

Fig. S8 Three-dimensional fluorescence spectra (top) and the corresponding contour plots (bottom) of free HSA (2.6 μM) and HSA bound to **C4** (2.6 μM) in 10 mM PBS (pH 7.4)

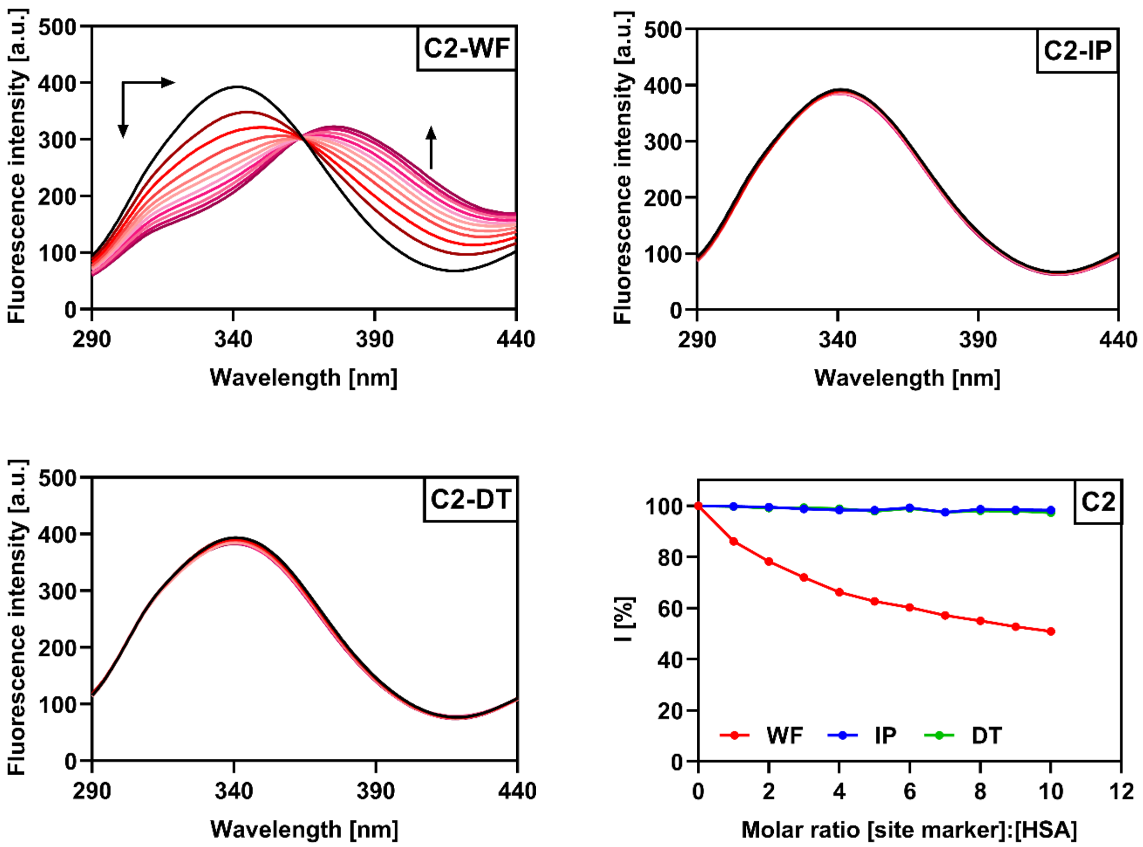

Fig. S9 Emission spectra of HSA-**C2** (1:1) complexes in 10 mM PBS (pH 7.4) upon addition of site markers warfarin (WF), ibuprofen (IP) and digitoxin (DT) along with a graphical analysis of the competitive experiment. Molar ratio of HSA-**C2**:site marker was 1:1, 1:2, 1:3, 1:4, 1:5, 1:6, 1:7, 1:8, 1:9 and 1:10

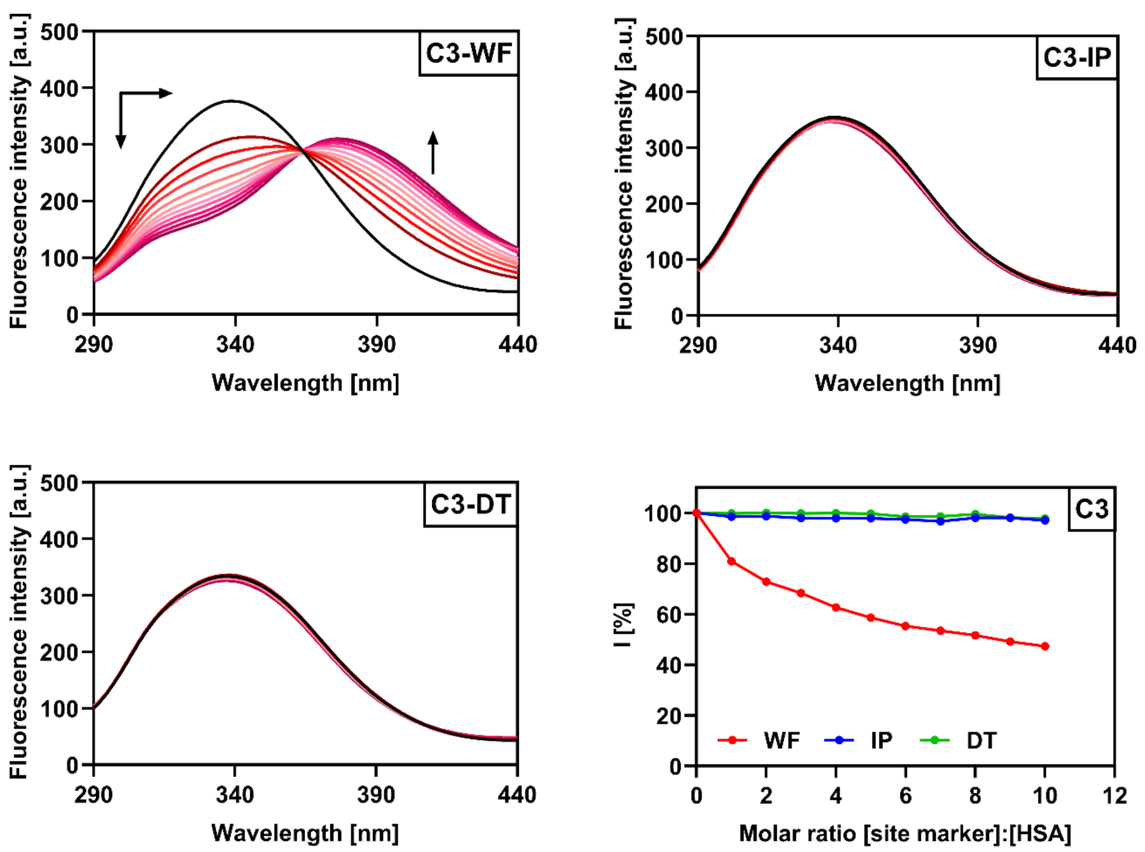

Fig. S10 Emission spectra of HSA-**C3** (1:1) complexes in 10 mM PBS (pH 7.4) upon addition of site markers warfarin (WF), ibuprofen (IP) and digitoxin (DT) along with a graphical analysis of the competitive experiment. Molar ratio of HSA-**C3**:site marker was 1:1, 1:2, 1:3, 1:4, 1:5, 1:6, 1:7, 1:8, 1:9 and 1:10

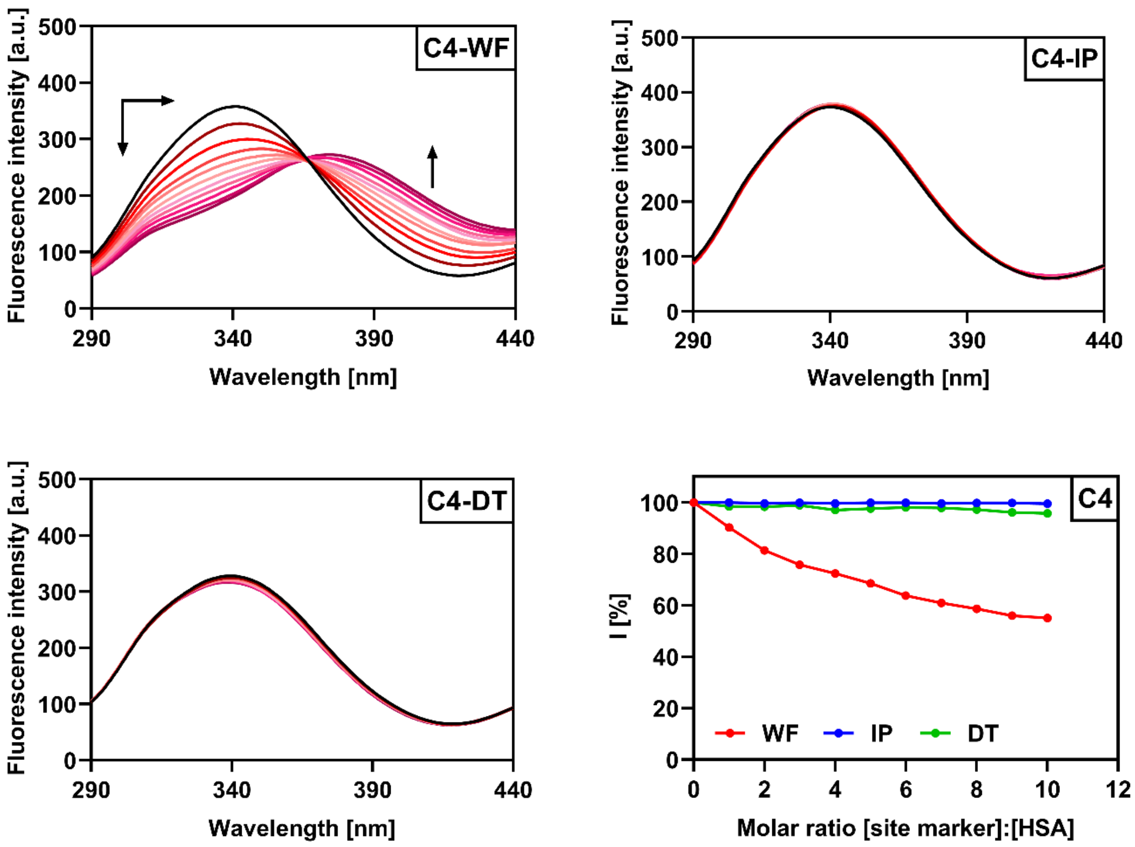

Fig. S11 Emission spectra of HSA-**C4** (1:1) complexes in 10 mM PBS (pH 7.4) upon addition of site markers warfarin (WF), ibuprofen (IP) and digitoxin (DT) along with a graphical analysis of the competitive experiment. Molar ratio of HSA-**C4**:site marker was 1:1, 1:2, 1:3, 1:4, 1:5, 1:6, 1:7, 1:8, 1:9 and 1:10

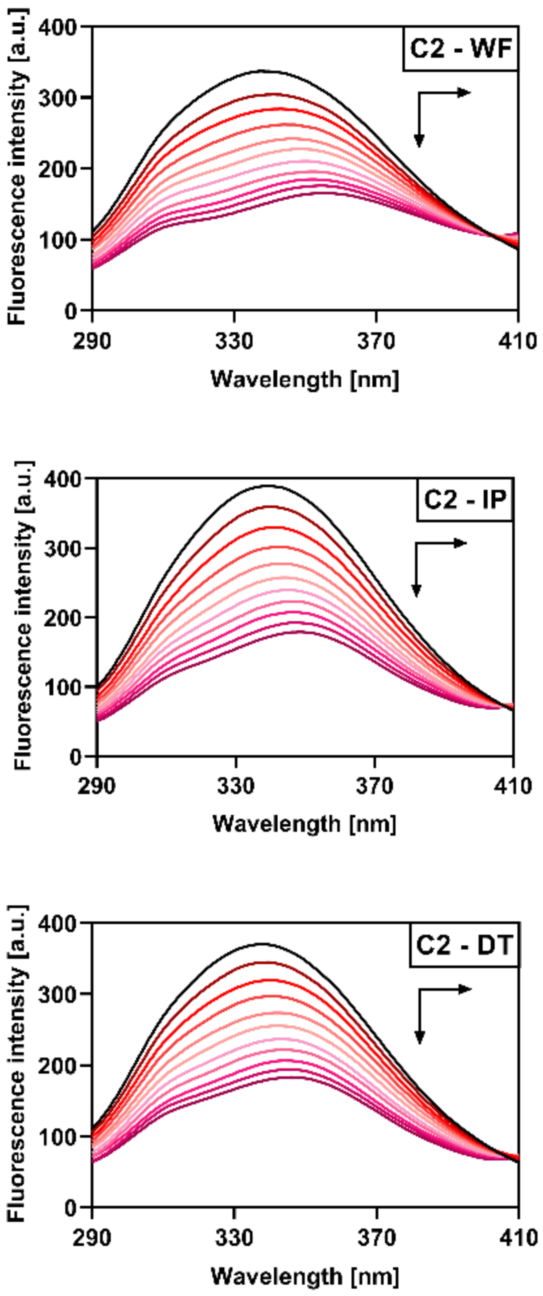

Fig. S12 Emission spectra of HSA-site marker (1:1) complexes in 10 mM PBS (pH 7.4) upon addition of **C2** (0-9.9 μM)

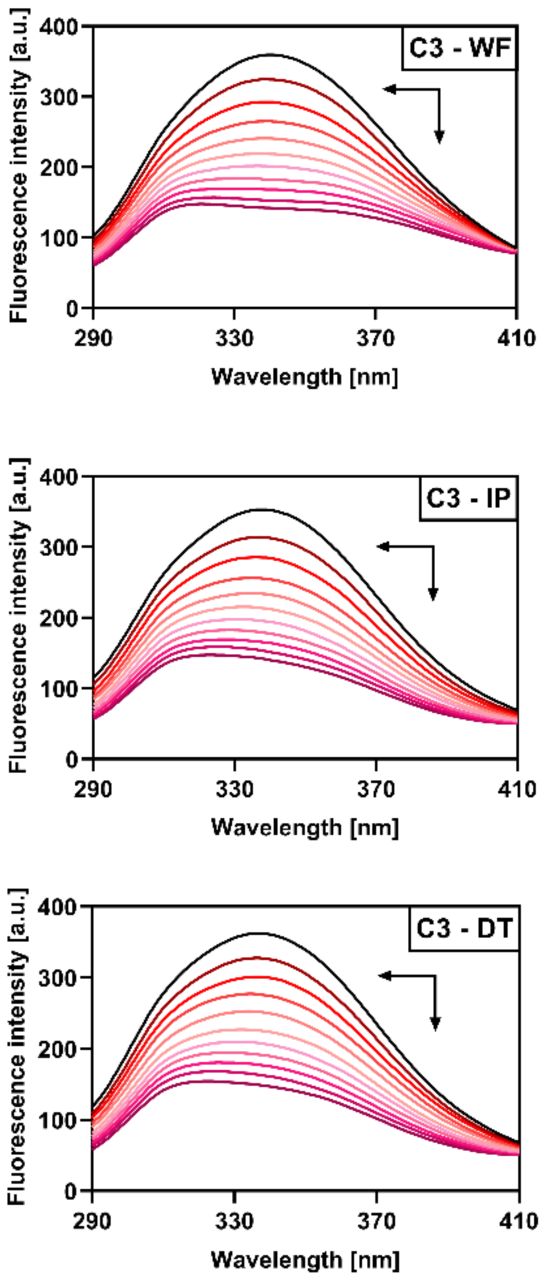

Fig. S13 Emission spectra of HSA-site marker (1:1) complexes in 10 mM PBS (pH 7.4) upon addition of **C3** (0-9.9 μM)

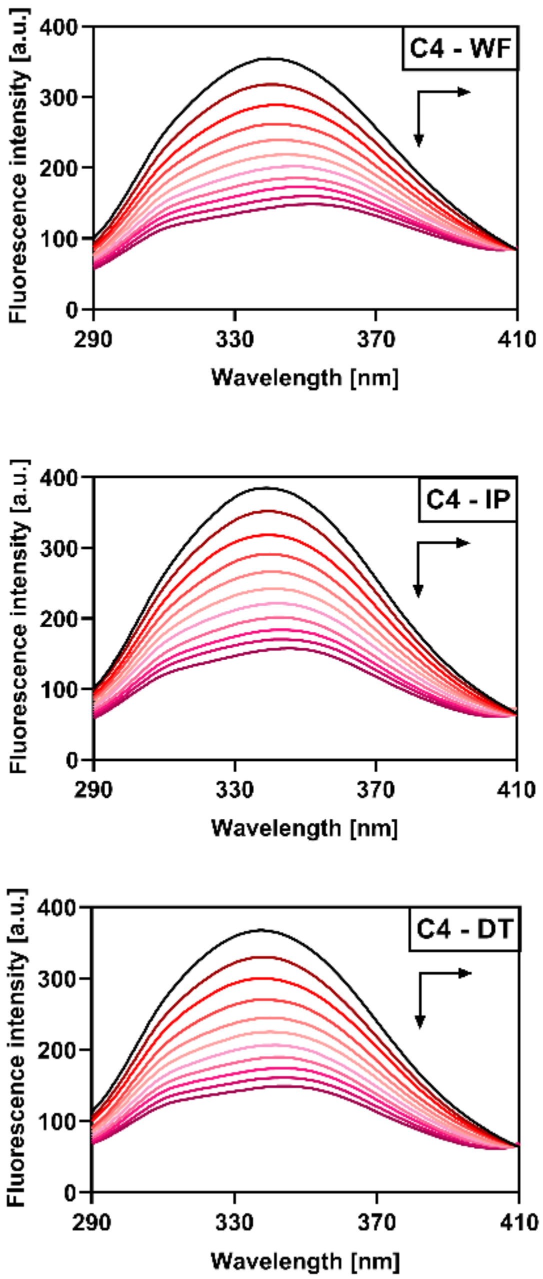

Fig. S14 Emission spectra of HSA-site marker (1:1) complexes in 10 mM PBS (pH 7.4) upon addition of **C4** (0-9.9 μM)

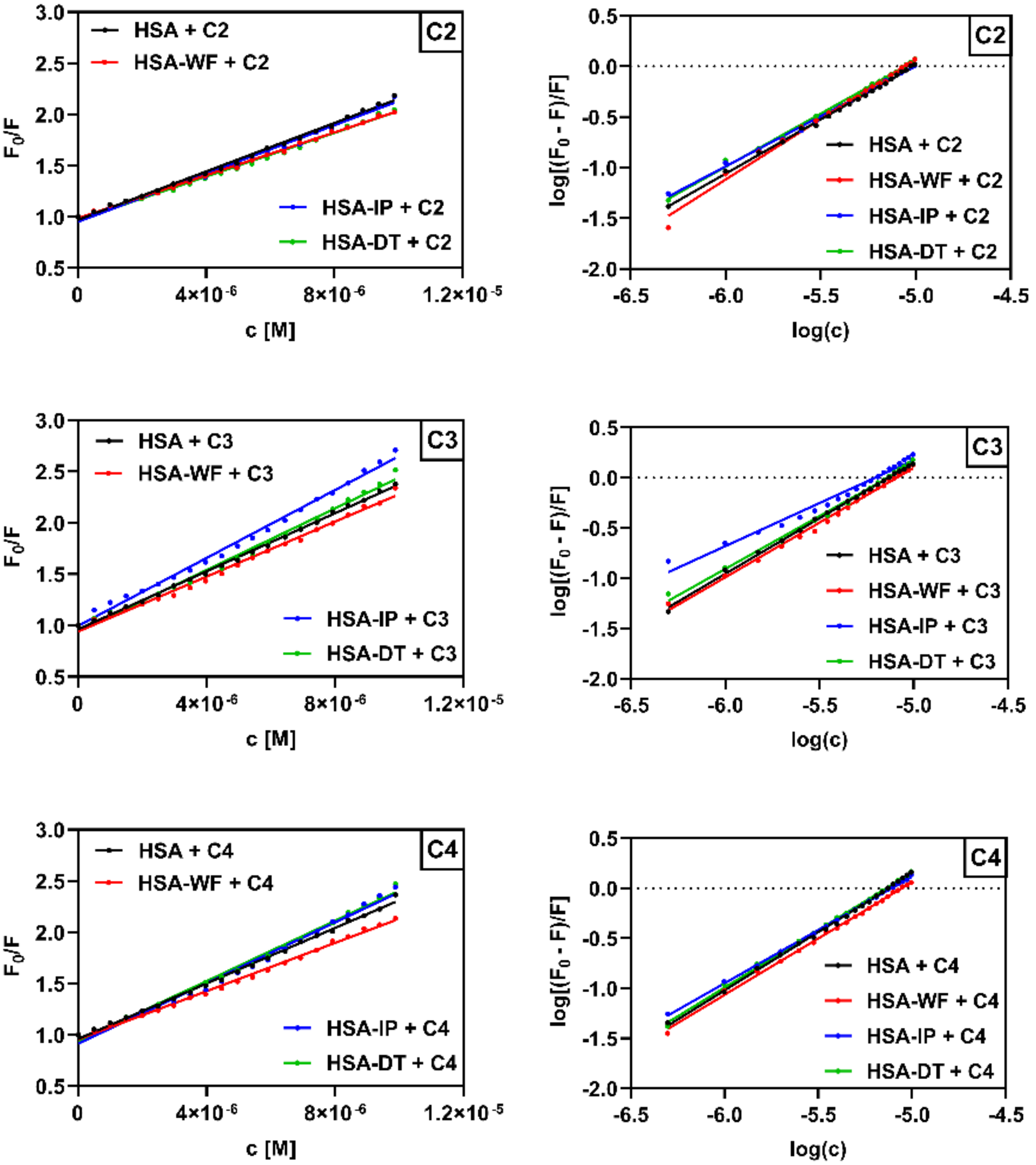

Fig. S15 Stern-Volmer plots (left) and logarithmic Stern-Volmer plots (right) for the fluorescence quenching of HSA-site marker (1:1) complexes by compounds **C2**-**C4** (0-9.9 µM)

Table S1 Predicted free energies of binding for the tested compounds with the hTopo I-DNA complex (PDB ID: 1T8I)

Compound	Free energy of binding[a] ($kcal.mol^{-1}$)	Free energy of binding[a] ($kJ.mol^{-1}$)
**C1**	-10.00	-41.87
**C2**	-9.98	-41.78
**C3**	-9.34	-38.89
**C4**	-9.29	-39.10

[a] for T = 293.15 K

Table S2 Amino acid residues involved in the interactions between the tested compounds and HSA (PDB ID: 1AO6)

Compound	Hydrogen bonds		Hydrophobic bonds		
	classical	non-classical	π-alkyl	π-π sigma	π-π stacked
**C1**	Phe134, Arg114, Leu115, Lys519	Leu115	Lys137	Leu115	-
**C2**	Asp129, Glu141, Tyr161	Met123	Leu115, Lys137	Ala126	-
**C3**	Leu115, Arg117, Phe134, Tyr138, Arg186	Pro118, Leu182	Lys 137, Leu182, Arg186	Ile142	Tyr138, Leu185
**C4**	Arg117, Phe134, Tyr161	-	Leu115, Arg117, Lys 137, Leu182	-	Phe134
